# *Primula vulgaris* (primrose) genome assembly, annotation and gene expression, with comparative genomics on the heterostyly supergene

**DOI:** 10.1038/s41598-018-36304-4

**Published:** 2018-12-18

**Authors:** Jonathan M. Cocker, Jonathan Wright, Jinhong Li, David Swarbreck, Sarah Dyer, Mario Caccamo, Philip M. Gilmartin

**Affiliations:** 1 School of Biological Sciences, University of East Anglia, Norwich Research Park, Norwich, NR4 7TJ United Kingdom; 2 Earlham Institute, Norwich Research Park, Norwich, NR4 7UZ United Kingdom; 3 National Institute for Agricultural Botany, Huntingdon Road, Cambridge, CB3 0LE United Kingdom

## Abstract

*Primula vulgaris* (primrose) exhibits heterostyly: plants produce self-incompatible pin- or thrum-form flowers, with anthers and stigma at reciprocal heights. Darwin concluded that this arrangement promotes insect-mediated cross-pollination; later studies revealed control by a cluster of genes, or supergene, known as the *S* (*Style length*) locus. The *P*. *vulgaris S* locus is absent from pin plants and hemizygous in thrum plants (thrum-specific); mutation of *S* locus genes produces self-fertile homostyle flowers with anthers and stigma at equal heights. Here, we present a 411 Mb *P*. *vulgaris* genome assembly of a homozygous inbred long homostyle, representing ~87% of the genome. We annotate over 24,000 *P*. *vulgaris* genes, and reveal more genes up-regulated in thrum than pin flowers. We show reduced genomic read coverage across the *S* locus in other *Primula* species, including *P*. *veris*, where we define the conserved structure and expression of the *S* locus genes in thrum. Further analysis reveals the *S* locus has elevated repeat content (64%) compared to the wider genome (37%). Our studies suggest conservation of *S* locus genetic architecture in *Primula*, and provide a platform for identification and evolutionary analysis of the *S* locus and downstream targets that regulate heterostyly in diverse heterostylous species.

## Introduction

**Figure 1:**
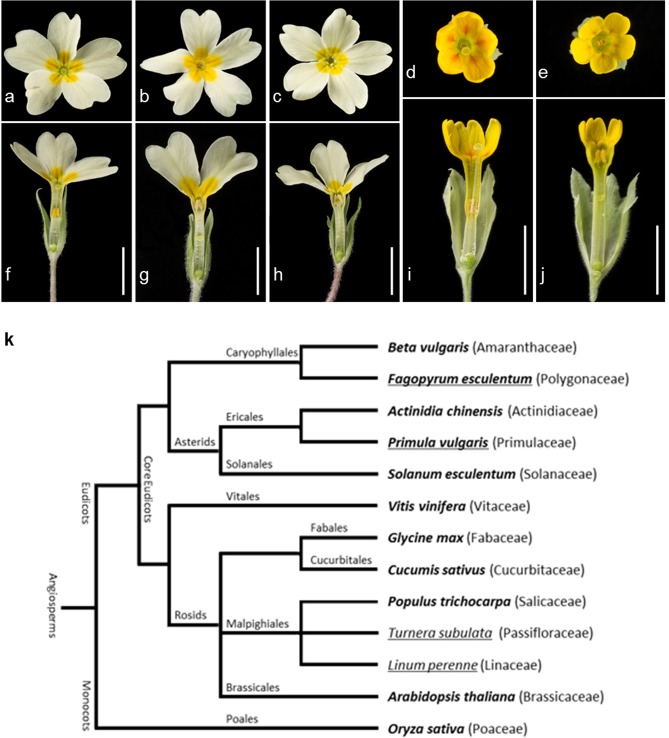
*Primula* floral phenotypes and angiosperm phylogeny. Floral phenotypes showing face and longitudinal flower sections of *P*. *vulgaris* pin (**a**,**f**), thrum (**b**,**g**) and long homostyle (**c**,**h**); *P*. *veris* pin (**d**,**i**) and thrum (**e**,**j**); scale bar, 1 cm. Schematic phylogeny of angiosperm species (**k**) adapted from https://genomevolution.org/wiki/index.php/Sequenced_plant_genomes#Phylogenetic_Tree and http://tolweb.org. Heterostylous species are underlined, species with sequenced and assembled genomes shown in bold.

Floral heteromorphy in *Primula* has been studied for over 150 years. Charles Darwin first recognized the importance of this breeding system for promoting cross-pollination^1,2^; observations on its existence date back even further^3^. In heterostylous *Primula* species, plants produce one of two forms of flower, pin or thrum, with anthers and stigma in reciprocal positions (Fig. 1). This arrangement physically promotes insect-mediated outcrossing between the two floral morphs. Pin flowers present the stigma at the mouth of the corolla tube, and the anthers halfway down (Fig. 1a,f,d,i). Thrum flowers present anthers at the mouth of the flower, and the stigma halfway down (Fig. 1b,g,e,j). In most *Primula* species, this physical mechanism functions alongside a pollen-pistil recognition self-incompatibility (SI) system, which inhibits self-fertilization^4–6^.

Darwin investigated the effects of cross and self-fertilization in numerous species^7^; he observed reduced seed set from within-morph crosses of *Primula*, and proceeded to define heterostyly as a mechanism to ensure outcrossing, and avoid the potential ill-effects of inbreeding on height, vigour and fertility^2^. This remarkable floral innovation is a striking example of convergent evolution, having evolved independently on at least 23 occasions, in 28 angiosperm families^8,9^. Heterostylous *Primula* species dominate the Primulaceae family, which resides in the order Ericales of the asterids lineage (Fig. 1k). Development of features specific to each floral morph (heterostyly) is controlled by the *S (Style length)* locus^5,10^, which is distinct from the self-incompatibility (*S*) locus described in a number of homomorphic self-incompatible species^11^. *Primula vulgaris* and the closely-related *P*. *veris* have 11 chromosome pairs (2n = 22). *P*. *vulgaris* has a predicted genome size by flow-cytometry of 459 Mb^12^ or 489 Mb^13^, giving a mean of 474 Mb, which is comparable to 479 Mb predicted for *P*. *veris*^13^; a *P*. *veris* genome assembly covering ~65% of the 479 Mb genome^14^ allowed differential expression analysis of a limited set of genes and cross-species comparisons, but did not facilitate assembly of the complete *S* locus.

Other commonly studied distylous genera include *Fagopyrum*^15^, *Turnera*^16^ and *Linum*^17^ (Fig. 1k). The role of heterostyly is an important consideration in attempts to produce true-breeding (homozygous) cultivars of *Fagopyrum esculentum* (buckwheat) for example: an assembly of the *F*. *esculentum* genome was recently reported^18^, with a view to identifying genes of agronomic significance in this food crop, such as those at the *S* locus. *Primula* are important horticultural (ornamental) crops in Europe, the United States and Japan^19^. For *Primula sieboldii*, genetic maps have been constructed to facilitate both conservation studies and the identification of genes underpinning the impressive floral variety of this species^20^. The availability of a high coverage *P*. *vulgaris* genome sequence will facilitate generation of an increased number of markers associated with favourable traits in horticultural cultivars, and also accelerate investigations into the developmental control and convergent evolution of heterostyly.

Despite the historical role of *Primula* species in establishing modern genetic theory^10,21–24^, and a range of heterostyly studies in different angiosperm families^15–17,25,26^, the molecular basis of the phenomenon has remained elusive. However, we recently reported the complete sequence of the *P*. *vulgaris S* locus as a thrum-specific genomic region comprising a cluster of five genes (expressed only in thrum)^27^; it is absent from pin, and hemizygous in thrums, not heterozygous as previously assumed^5,6^. Occasionally, homostyle *Primula* plants arise, with anthers and stigma at the same height^5,6,27^. These were thought to arise through recombination between dominant and recessive *S* locus alleles in thrum plants^5,6^. However, the hemizgous architecture of the *S* locus precludes such recombination, which suggests that self-fertile homostyle plants must arise through mutation of *S* locus genes. *GLO*^*T*^ and *CYP*^*T*^ were identified as the genes controlling anther elevation and style length, respectively, based on such mutations^27^. Others have described the function of *CYP*^*T*^ (CYP734A50)^28^, and confirmed our finding that *CYP*^*T*^ lies adjacent to *GLO*^*T*^ ^29^. The *S* locus gene *GLO*^*T*^ is an apparent duplication of *PvGLO*, the *P*. *vulgaris* orthologue of the B-function MADS-box gene *GLOBOSA*^30^. Our phylogenetic studies revealed an estimated divergence of 51.7 MYA for *GLO*-*GLO*^*T*^^27^, which suggests a single origin for heterostyly in the Primulaceae, and led us to predict that the *S* locus structure might be shared amongst *Primula* species.

Here, we present an annotated *P*. *vulgaris* genome assembly representing ~87% of the predicted 474 Mb *P*. *vulgaris* genome^12,13^. Our assembly is based on a *P*. *vulgaris* long homostyle plant with high genome-wide homozygosity. We previously reported the 278 kb *S* locus in *P*. *vulgaris* as a hemizygous complex locus: this region was generated using contigs from our long homostyle, thrum and BAC assemblies^26,27^. These previous findings raised new questions that we explore in the current manuscript: we address whether the hemizygosity of the *S* locus and its constituent genes is conserved in different *Primula* species; given this, we examine genomic features in the non-recombining *S* locus to determine how unique the region is compared to the wider genome; and, we explore genome-wide differential gene expression, using the comprehensive geneset defined in our *P*. *vulgaris* genome assembly, to identify potential direct and indirect downstream targets of this regulatory locus. Our *P*. *vulgaris* genome assembly provides a resource for future anchoring to chromosomes, analysis of genome-wide gene families and downstream targets, and investigations on the evolution and function of heterostyly across the Primulaceae and other angiosperm families.

## Results

### *Primula vulgaris* genome assembly

To generate the *P*. *vulgaris* genome assembly (LH_v2), we selected a long homostyle plant originating from the Somerset Wyke Champflower population^31,32^. *P. vulgaris* homostyle plants (Fig. 1c,h) are self-fertile^33^, producing offspring with greater allelic homozygosity than out-crossed pin or thrum plants. In addition to the opportunity for self-pollination in the natural population, this line underwent further rounds of selfing in cultivation.

**Figure 2:**
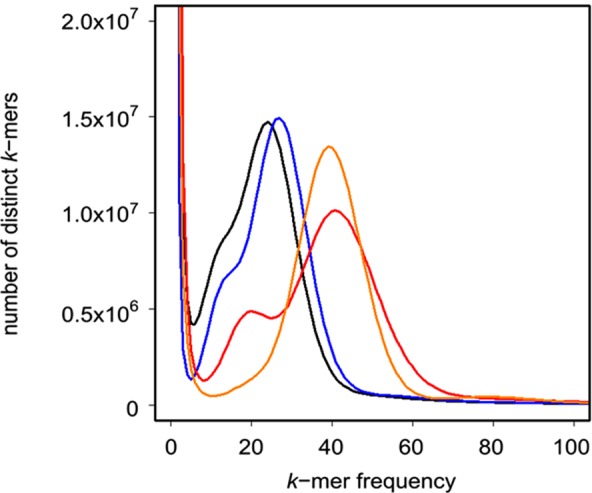
*P*. *vulgaris* genomic sequencing reads *k*-mer plot. *K*-mer frequency-abundance distribution (*k* = 31) generated from *P*. *vulgaris* paired-end sequencing reads: long homostyle (Somerset) (orange), long homostyle (Chilterns) (blue), thrum (red), and pin (black).

We analysed the *k*-mer frequency-abundance distribution of genomic paired-end reads (Illumina HiSeq) (Supplementary Table S1) from the *P*. *vulgaris* long homostyle (Somerset) plant (Fig. 2). Supplementary Tables S1–S3 contain read libraries corresponding to (unless indicated) Supplementary Table S1a in our previous *S* locus studies^27^, and are included here for convenience and completeness. The long homostyle (Somerset) data reveal a unimodal distribution beyond the first local minima, characteristic of a homozygous genome^34^. *K*-mer frequency-abundance distributions for *P*. *vulgaris* pin and thrum (Fig. 2) show notable secondary peaks, indicating heterozygosity. We also obtained a second long homostyle from the Chiltern population using maps drawn by Crosby^35^. However, *k*-mer analysis reveals the genome of this individual is more heterozygous than the Somerset homostyle (Fig. 2), suggesting more recent outcrossing. The greater homozygosity of the Somerset long homostyle over heterozygous pin and thrum plants, and the Chiltern long homostyle, underpins our *P*. *vulgaris* genome assembly.

We assembled genomic short read and mate pair sequences from the Somerset *P*. *vulgaris* long homostyle (Supplementary Table S1) using SOAPdenovo v2.04^36^. Removal of 3,516 non-*Primula* contigs resulted in 577,740 scaffolds representing 481.3 Mb of sequence with a scaffold-N50 of 236.3 kb; after removal of scaffolds <200 bp in length, the assembly comprised 411.2 Mb of sequence in 67,619 scaffolds. Further processing identified 128 contigs containing chloroplast sequences from *Primula* species (GenBank accessions KU321892.1, KF753634.1, and KX639823.1), resulting in a final assembly (LH_v2) of 67,491 contigs covering 411.1 (411) Mb (Table 1). We also generated a draft 441.5 Mb *P*. *veris* thrum assembly (VT_v1) using paired-end reads only (Supplementary Table S1) (n = 145,617; N50 10.8 kb; NG50 9.5 kb; contigs ≥200 bp). The 411 Mb *P*. *vulgaris* LH_v2 assembly (N50 294.8 kb, NG50 229.8 kb) represents 87% of the estimated 474 Mb genome based on the mean of two flow cytometry estimates for *P*. *vulgaris* genome size, 459 Mb^12^ and 489 Mb^13^; the NG50 was calculated (conservatively) using the higher of these two estimates. NG50 is a more robust metric than N50 that considers the estimated genome size rather than the size of the generated assembly^37,38^. LH_v2 represents a substantial improvement in contiguity and completeness over a previously published 309.7 Mb *P*. *veris* assembly^14^, which covers 65% of the estimated 479 Mb *P*. *veris* genome^13^ (N50 165.8 kb, NG50 73.3 kb) (Supplementary Fig. S2). The published *P*. *veris* assembly^14^ contains 40.7 Mb (13.14%) “N”s (ambiguous bases) compared to 29.9 Mb (7.26%) in the 411 Mb *P*. *vulgaris* assembly.

**Table 1:** Genome assembly statistics for *P*. *vulgaris* LH_v2 and gene annotations at various stages of the assembly process.

	SOAP contigs	SOAP scaffolds	Scaffolds (gap filled)	Scaffolds ≥200 bp*
Total	1,787,577	581,256	581,256	67,491
Total size (Mb)	581.3	497.8	482.0	411.1
Ns (%)	0	21.0	6.2	7.3
N50 (kb)	1.0	249.8	236.1	294.8
NG50 (kb)	1.1	256.0	230.0	229.8
Max length	53 kb	1.7 Mb	1.6 Mb	1.6 Mb
Repeat (%)	—	—	41.63	37.03
Gene loci	—	—	26,116	24,599
Alternative spliceoforms	—	—	4,488	4,488
Mean CDS length (bp)	—	—	1,401	1,466

^*^Contaminated contigs removed.

Genome statistics for *P. vulgaris* LH_v2 contigs, scaffolds, gap-filled scaffolds, and gap-filled scaffolds ≥200 bp: only the gap-filled scaffolds were annotated. The final assembly (“Scaffolds ≥200 bp”) comprises scaffolds ≥200 bp with non-*Primula* and chloroplast containing scaffolds removed. Ns = ambiguous bases.

For assembly validation, we generated *k*-mer copy number plots (Supplementary Fig. S1). These figures show that the vast majority of *k*-mers in the genomic sequencing reads are present in the assembly whilst low-frequency *k*-mers expected to represent sequencing errors, are not. There is minimal change to the observed *k*-mer spectra when removing contaminated contigs (Supplementary Fig. S1b,c) or contigs <200 bp (Supplementary Fig. S1c,d), suggesting these steps did not result in significant loss of genomic content. RNA-Seq reads (Supplementary Table S2) aligned to the assembly for gene prediction (see below) produced mean overall and concordant pair alignment rates of 91.1% and 85.0%. Furthermore, the assembly includes 97.2% of 248 Core Eukaryotic Genes (CEGs) expected to be present in the majority of eukaryote genomes^39^ (Supplementary Table S4). These analyses suggest that most of the *P*. *vulgaris* genespace has been captured in the assembly.

### Repeat sequences in the *Primula* genome and *S* locus

To analyse repeat sequence composition of the *P*. *vulgaris* genome we generated a *de novo* repeat library which revealed 37% of the assembled 411 Mb *P*. *vulgaris* genome as repetitive; classification of repeat sequences in the LH_v2 genome is detailed in Supplementary Table S5. TEs (transposable elements) comprise over 35% of the genome, which is comparable to the predicted TE content (>35%) in the assembly of the ~389 Mb *Oryza sativa* (rice) genome^40^, as well as the 758 Mb assembly of the more closely-related *Actinidia chinensis* (kiwifruit) (36%)^41^.

We used our *de novo* repeat library to annotate the contiguous 278 kb *P*. *vulgaris S* locus^27^. This revealed the region as particularly rich in TEs (64%), in the top 5% compared to both genome-wide contigs (>10 kb) (Supplementary Fig. S4a), and similarly-sized contigs (278 kb ± 20%) (Supplementary Fig. S4b). In contrast, analysis of 171 kb of sequence flanking the *S* locus^27^ does not reveal an elevated repeat content (34.67%) (Supplementary Fig. S4a). For the published *P*. *veris* genome^28^, the Repbase library^42^ was used to annotate 7.7% of the published assembly as repetitive^14^. Our comprehensive *P*. *vulgaris* repeat library enabled us to annotate 25% of the published *P*. *veris* assembly^14^, and 35% of our draft *P*. *veris* (VT_v1) thrum assembly (contigs <200 bp removed), as repetitive.

### *P*. *vulgaris* gene annotation

Our *P*. *vulgaris* LH_v2 genome assembly was annotated using RNA-Seq datasets from five tissues (Supplementary Table S2) to predict a total of 24,599 genes; these comprise 29,087 coding sequences, with 4,488 recognised as alternative splice variants. Functional descriptions were assigned to ~85% of genes based on homology to SwissProt, TrEMBL (http://www.uniprot.org/) and TAIR10 (https://www.arabidopsis.org/) protein databases. Of these, ~90% contain at least one domain, and ~60% are annotated with Gene Ontology (GO) terms. OrthoMCL analysis identified 19,861 orthologous gene groups for *P*. *vulgaris* compared to five angiosperm species (Supplementary Fig. S3).

To investigate the accuracy of the predicted *P*. *vulgaris* gene count, we compared coding sequences in *P*. *vulgaris* (24,599) and *P*. *veris* (18,301)^14^ (Fig. S3c). We reveal 1,166 *P*. *vulgaris* coding sequences absent from the *P*. *veris* assembly, and 6,501 *P*. *vulgaris* coding sequences absent from *P*. *veris* gene annotations; the reciprocal analysis reveals 685 *P*. *veris* coding sequences absent from the *P*. *vulgaris* geneset, and 130 coding sequences absent from *P*. *vulgaris* contigs. RNA-Seq reads used for *P*. *veris* gene prediction, produced mean overall and concordant pair alignment rates of 82.5% and 75.7% respectively when mapped to the *P*. *veris* genome assembly, compared to 91.1% and 85.0% for *P*. *vulgaris* RNA-Seq reads mapped to LH_v2.

### *P*. *vulgaris* RNA-Seq expression analysis

**Figure 3:**
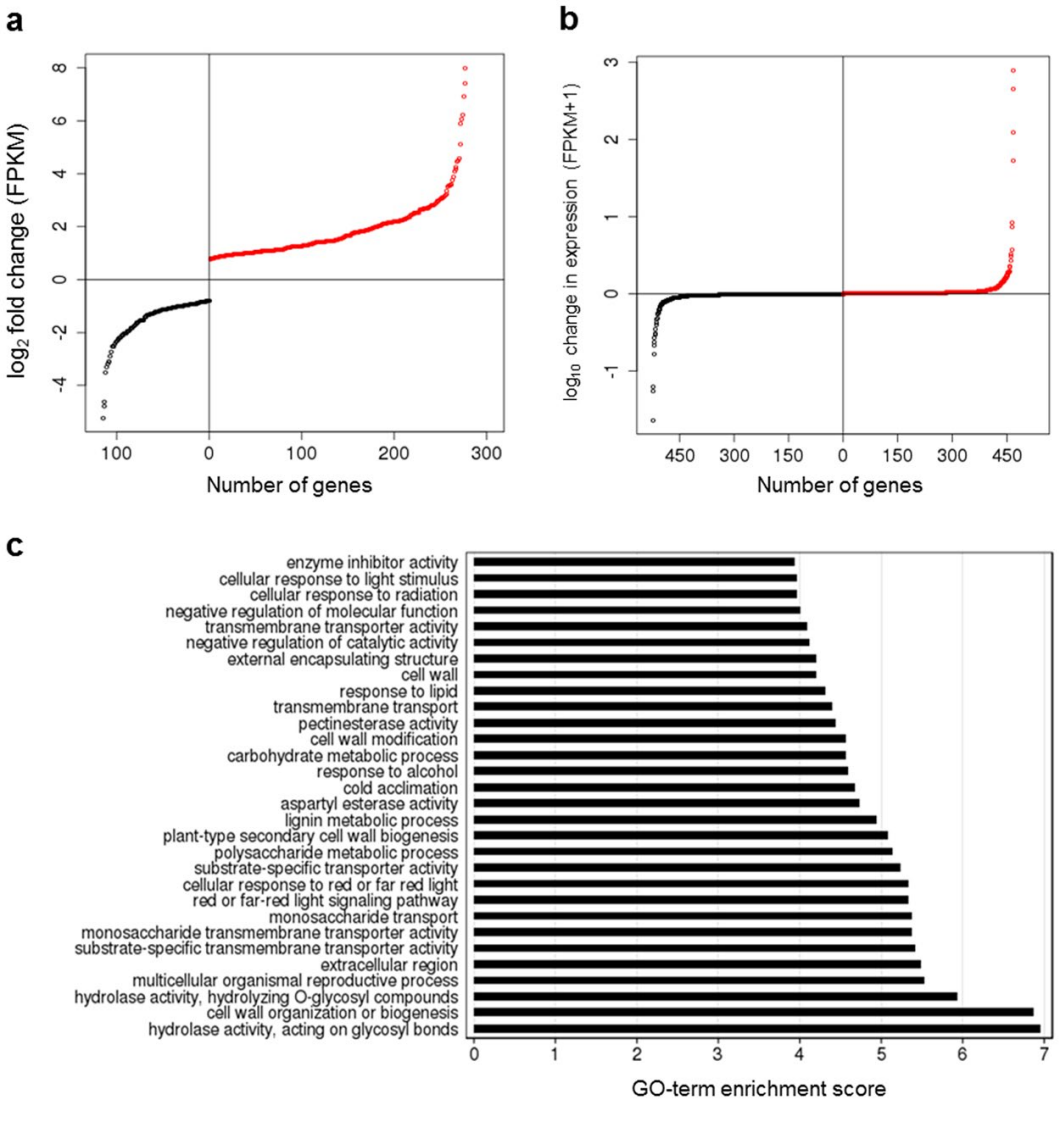
*P*. *vulgaris* pin and thrum flower differential gene expression, and GO term enrichment. (**a**) Genes with significantly higher expression (<0.05 FDR) in thrum (upregulated in thrum) (n = 283) (red), or pin (downregulated in thrum) (n = 118) (black) flowers, with log_2_ fold-change in expression in thrum compared to pin shown (FPKM, Fragments Per Kilobase of transcript per Million fragments mapped); genes expressed in only one morph are excluded; (**b**) Genes expressed in only one morph: log_10_ change in expression (FPKM + 1) in response to the presence of the *S* locus in thrum; red = genes upregulated in thrum flowers (expressed in thrum only) (n = 525); black = genes downregulated in thrum flowers (expressed in pin only) (n = 468); (**c**) Gene Ontology (GO) terms assigned to the 401 significantly differentially expressed genes (a), and their associated GO-term enrichment scores (top 30 over-represented GO terms (False Discovery Rate (FDR) < 0.1) are shown); enrichment score = −log_10_(uncorrected p-value), with the uncorrected p-value as calculated in GO-term enrichment analysis versus GO term occurrence in *P*. *vulgaris* LH_v2 functional annotations.

We carried out RNA-Seq differential expression analysis using the full complement of predicted *P*. *vulgaris* genes analysed above as a guide. RNA-Seq reads were generated using 15–20 mm floral buds from four pin plants and four thrum plants (four biological replicates) (Supplementary Table S3); these plants were siblings from a controlled cross in the same population, which we reasoned would control for individual background variation. The analysis revealed 401 genes expressed in both pin and thrum flowers at different levels: 283 genes were significantly up-regulated, and 118 genes significantly down-regulated, in thrum flowers as compared to pin (FDR < 0.05) (Fig. 3a) (see http://opendata.earlham.ac.uk/primula for gene sequences). Four of the five thrum-specific *S* locus genes (*CYP*^*T*^, *PUM*^*T*^, *KFB*^*T*^, *CCM*^*T*^)^27^ show low expression compared to the differentially expressed geneset (Supplementary Fig. S5). We also identified 525 genes expressed uniquely in thrum flowers and 468 genes expressed uniquely in pin flowers (Fig. 3b). For the 401 differentially expressed genes, GO-term enrichment analysis relative to GO term frequency in the full *P*. *vulgaris* geneset revealed overrepresentation of GO terms potentially related to cell wall modification and reproductive processes (Fig. 3c). In contrast, despite a small number of genes showing relatively high expression, including the *S* locus gene *GLO*^*T*^ (log_10_ FPKM + 1 = 1.72) (Supplementary Fig. S5), there were no over-represented GO terms in the morph-specific (pin or thrum only) expression dataset (Supplementary Table S6); this was also true using >0.1 FPKM or >1 FPKM minimum cutoffs for expression.

### Genetic architecture of the *S* locus in *Primula* species

**Figure 4:**
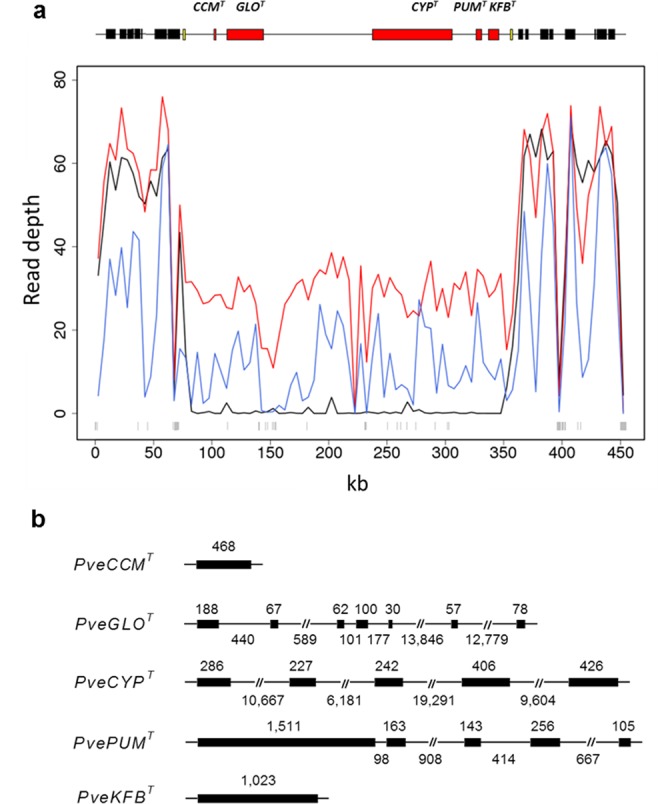
*Primula veris S* locus genetic architecture and gene structures. (**a**) Genomic read depth across the 455 kb *P*. *vulgaris S* locus assembly region in 5 kb non-overlapping windows: *P*. *veris* thrum (blue), *P*. *vulgaris* thrum (red) and pin (black); grey vertical lines above the x-axis represent ambiguous bases (“N”s) in the assembled sequence. The schematic above the graph shows the approximate size and location of *P*. *vulgaris* genes^27^ within this region: thrum-specific *S* locus genes (red), tandem-repeat *CFB* loci that flank the 278 kb *S* locus (yellow), and predicted genes flanking the *S* locus (black) are indicated. (**b**) gene structures of *P*. *veris* (*Pve*) orthologues of the five genes identified in the *P*. *vulgaris S* locus^27^. Exons (thick lines) and introns (thin lines; approximate size) are shown; introns >0.5 kb are displayed as 0.5 kb (see Supplementary Fig. S4 for expanded intron size schematic).

The *P*. *vulgaris* long-homostyle genome assembly generated in this project provided four contigs that facilitated our previous definition of the *S* locus as a 278 kb thrum-specific (hemizygous) region^27^, comprising a cluster of five genes present in thrum (*GLO*^*T*^, *CYP*^*T*^, *PUM*^*T*^, *KFB*^*T*^, *CCM*^*T*^) but absent in pin (Fig. 4a). Here, we examine whether the hemizygous genetic architecture is conserved in other *Primula* species. We mapped our genomic paired-end sequence reads from an individual *P*. *veris* thrum plant (Supplementary Table S1) (Fig. 4a) to the *P*. *vulgaris* genome assembly incorporating the previously identified complete 455 kb assembly^27^ of the *P*. *vulgaris S* locus (278 kb), flanking regions (171 kb), and flanking *CFB* loci (~6 kb)^27^. Our results suggest that the *S* locus is also hemizygous in *P*. *veris*, revealed by a notable drop in coverage compared to the flanking regions (Fig. 4a). This suggests the hemizygous genetic architecture of the *Primula S* locus is an important feature that is evolutionary conserved. The *de novo* assembly of the complete *S* locus region in *P*. *veris* was not possible using published *P*. *veris* genome data^14^, or our *P*. *veris* thrum read library; alignments reveal a fragmented array of assembled contigs. However, the above genomic analyses demonstrate the utility of our *P*. *vulgaris S* locus assembly as a reference for investigating evolutionary conservation of the region in related *Primula* species.

**Figure 5:**
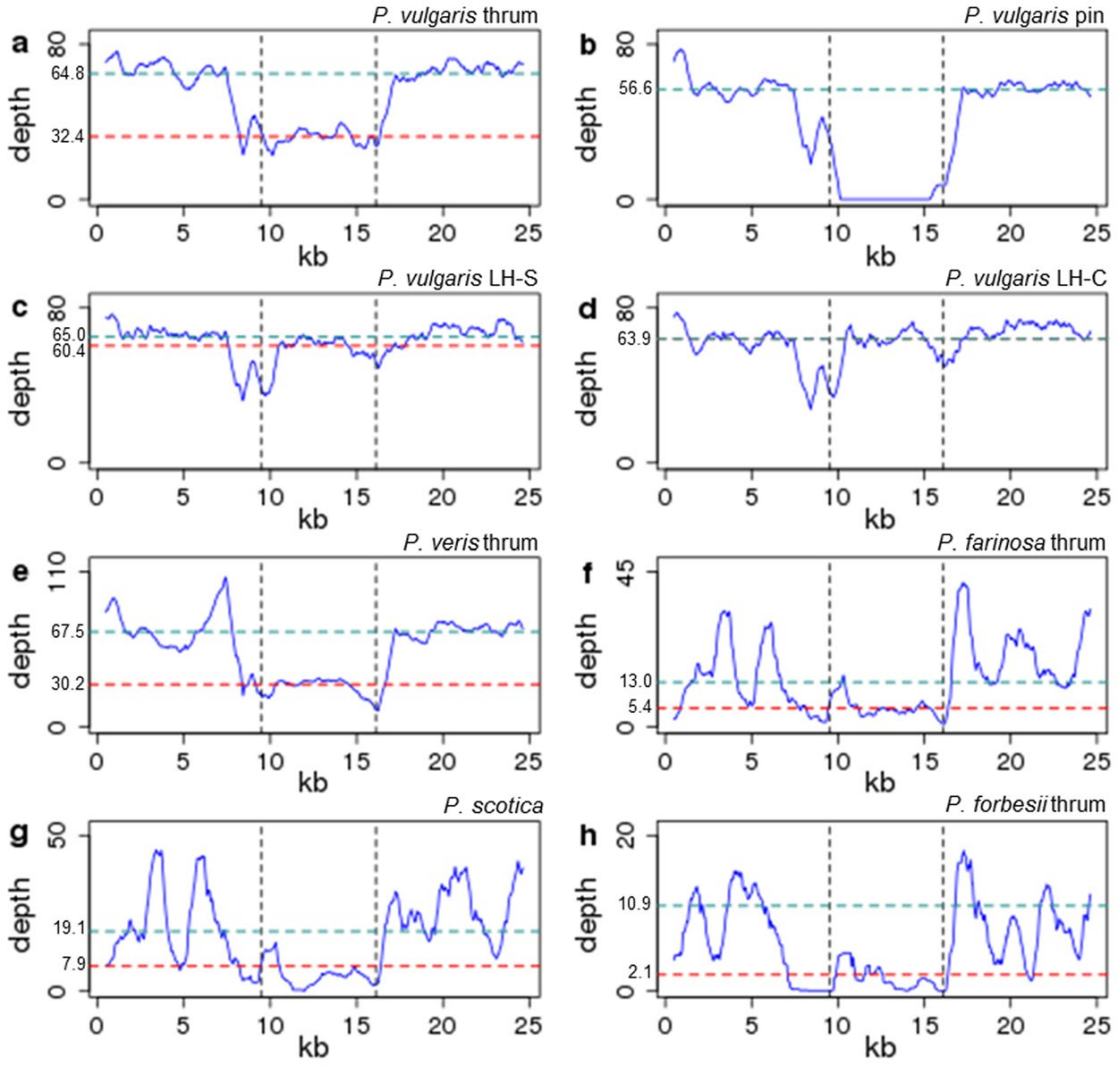
*Primula S* locus genomic read depth across coding sequence positions. Genomic read depth across predicted coding sequences (CDSs) in the 455 kb *S* locus and flanking regions in different *Primula* morphs and species: *P*. *vulgaris* thrum (*S* locus hemizygote) **(a)**, pin (*S* locus null) (**b**), long homostyle Somerset (LH-S) (*S* locus homozygote) (**c**), long homostyle Chiltern (LH-C) (*S* locus homozygote) (**d**); *P*. *veris* thrum (**e**); *P*. *farinosa* thrum (**f**); *P*. *scotica* (homostyle species) (**g**); *P*. *forbesii* thrum (**h**). Black vertical dotted lines define CDSs within the 278 kb *P*. *vulgaris* thrum-specific *S* locus. Horizontal dotted lines indicate median read depth for the *S* locus (red), and its flanking regions (blue), with these values labelled on the y-axis (small black text); these lines overlap for Chiltern long homostyle (d); *P*. *vulgaris* pin (b) has zero coverage across the *S* locus CDSs.

Further investigation of *S* locus genomic read depth was carried out for *P*. *vulgaris*, *P*. *veris*, and the more distantly related *P*. *farinosa*, *P*. *scotica* (Table S1), and *P*. *forbesii*^28^ (Fig. 5). To avoid read mismapping for distant species, we examined coding sequence regions defined by predicted LH_v2 gene models only. This analysis revealed reduced read depth (coverage) at the *S* locus compared to the flanking regions in *P*. *vulgaris* thrum (Fig. 5a), and zero coverage in *P*. *vulgaris* pin (Fig. 5b), as might be expected^27^. *P*. *vulgaris* homostyle plants originating from Somerset (Fig. 5c) and Chiltern (Fig. 5d) populations^31,32^ show diploid read coverage across the *S* locus. Both these homostyle mutants are self-fertile, and genetic analysis (not shown) reveals they are homozygous diploid for the *S* locus carrying *CYP*^*T*^ mutations. *P*. *veris* thrum shows reduced coverage at the *S* locus (Fig. 5e), as observed in *P*. *vulgaris* thrum^27^.

For *P*. *farinosa* thrum (Fig. 5f), *P*. *scotica* (homostyle species) (Fig. 5g) and *P*. *forbessii* thrum (Fig. 5h), there are fewer reads mapped to the *S* locus, but the coverage profile is nonetheless distinct from the zero coverage observed for *P*. *vulgaris* pin (Fig. 5b) from which the *S* locus is absent. Reduced coverage at the *S* locus for *P*. *farinosa* (diploid) and *P*. *forbesii* (assumed diploid) (Fig. 5f,g) suggests this region could also be hemizygous in thrum for these species, as in *P*. *vulgaris* and *P*. *veris*. Figure 5h was compiled using *P*. *forbesii* genomic read data from BioProject PRJNA317964^28^. Reduced read coverage to the left of the *S* locus may be due to similarity with other genomic regions, a result of “N”s (ambiguous bases) in surrounding sequences, or an incorrect gene prediction. *P*. *scotica* is a naturally occurring hexaploid homostyle species^43^; rather than showing comparable coverage within and outside the *S* locus as for the *P*. *vulgaris* homostyles (Fig. 5c,d) it displays reduced coverage within the central region (Fig. 5g), which might suggest *P*. *scotica* retains one or more pin chromosomes within its hexaploid karyotype. The above analyses reveal reduced coverage at the *S* locus in all *Primula* species tested.

### *Primula veris S* locus gene structures and expression

To determine whether the *S* locus gene sequences and structure are conserved in *P*. *veris*, genes previously identified at the *P*. *vulgaris S* locus^27^ were aligned to our draft *P*. *veris* thrum assembly (VT_v1) and the published *P*. *veris* genome^14^, alongside PCR analysis of *P*. *veris* cDNA. This analysis facilitated the definition of *P*. *veris* gene models for *PUM*^*T*^ (MF317488) and *CCM*^*T*^ (MF317489), and the correction of those for *GLO*^*T*^, *CYP*^*T*^ and *KFB*^*T*^ (MF317487) (Fig. 4b). The intron-exon structures of the *S* locus genes are conserved (Fig. 4b; Supplementary Fig. S4d).

We show expression for all five *P*. *vulgaris S* locus genes in *P*. *veris (Pve)* flowers, with PCR analysis of *P*. *veris* cDNA, and analysis of available RNA-Seq data using our curated gene models (above) as a guide for alignment^14,28^ (Supplementary Fig. S6). In the RNA-Seq dataset used for previous annotation of the published *P*. *veris* genome assembly (PRJNA238546)^14^, we reveal expression for four of the five *S* locus genes: three show thrum-specific expression, and one (*PveCYP*^*T*^) shows expression in thrum, with an extremely low number of pin flower RNA-Seq reads presumably erroneously mapped (Supplementary Fig. S6). *PveCCM*^*T*^ shows no expression in this dataset (Supplementary Fig. S6a); the gene was not annotated in the published *P*. *veris* genome assembly using this data, suggesting no transcript support in the previously reported single (unreplicated) RNA-Seq library at the sampled developmental stage^14^; we therefore manually annotated this gene to enable the analyses detailed here. The above findings prompted our PCR analysis of *P*. *veris* mixed-stage flower buds to confirm expression; this investigation revealed expression for all five genes (Supplementary Fig. S6b). Furthermore, our analysis of available RNA-Seq reads derived from corolla tubes and styles of *P*. *veris* pin and thrum flowers^28^ (pooled from 25 plants) (PRJNA317964) shows that all five genes are, as expected for this thrum-specific region, not expressed in pin for both these tissues; expression in one or more thrum plants serves as a positive control (Supplementary Fig. S6c). *GLO*^*T*^ was previously shown absent from 200 *P*. *veris* pin plants from a wild population^27^ (hence, pin RNA-Seq reads aligned to *PveGLO*^*T*^ are mapping errors). In conclusion, these analyses indicate the conserved presence and thrum-specific expression of all five *P*. *vulgaris S* locus gene homologues in *P*. *veris* thrum, alongside absence of expression in pin. This is consistent with the apparent hemizygosity of the region in *P*. *veris* thrum (Fig. 4a), and further demonstrates conservation of *S* locus features between these two *Primula* species.

Larger intron lengths and increased prevalence of TEs have been associated with regions showing reduced recombination rates in some eukaryotes^44^. The hemizygous genetic architecture of the *S* locus appears to be a conserved feature, which defines the region as non-recombining. Therefore, in addition to the TE content analysed above (Supplementary Fig. 4a,b), we investigated intron sizes at the *P*. *vulgaris S* locus compared to the wider genome. For this analysis, we examine all five *S* locus genes, using precise intron sizes for *PvGLO*^*T*^ and *PvCYP*^*T*^ that were previously determined using PCR and alignments to our draft *P*. *vulgaris* genome assemblies^27^; unambiguous sequence across corresponding introns is not available for *P*. *veris*. We previously reported the large introns of *CYP*^*T*^ and *GLO*^*T*^ in *P*. *vulgaris (Pv)*; *GLO*^*T*^ spans 25 kb with two introns over 10 kb; *CYP*^*T*^ spans 68 kb with 10, 20 and 30 kb introns^27^. *P*. *veris GLO*^*T*^ shares this feature (Supplementary Fig. S4d), as does *CYP*^*T*^ ^28^ (Supplementary Fig. S4d). *P*. *vulgaris GLO*^*T*^ and *CYP*^*T*^ introns >5 kb (median 11,463.5 bp, n = 6) are in the top 5% of intron lengths genome-wide; the median genome-wide intron size is 440 bp (n = 133,334) (Supplementary Fig. S4c). The median intron size in regions flanking the *S* locus is 351 bp (n = 91). The remaining introns, including both those <5 kb in *PvGLO*^*T*^ and *PvCYP*^*T*^, and those in *PvPUM*^*T*^, *PvKFB*^*T*^ and *PvCCM*^*T*^, are all <1 kb (median 426.5 bp, n = 8) (Supplementary Fig. S4c,d). There is greater intergenic distance (reduced gene density) between genes at the *P*. *vulgaris S* locus (10,925 bp, 103,811 bp, 17,438 bp, 13,307 bp; median 15,372.5 bp) (n = 4) compared to the flanking regions (median 1,816; n = 13), suggesting the absence of recombination might have impacted the genomic composition of the region.

## Discussion

*Primula vulgaris* exhibits floral heteromorphy (heterostyly), with reciprocal positioning of the anthers and stigma (Fig. 1a,b), and an SI system that prevents fertilization from pollen of the same floral morph. Together, these adaptations increase allelic heterozygosity in obligate outcrossing pin and thrum plants, which complicates the assembly of genomic short sequencing reads^45^ in this species. For genome assembly, the low allelic diversity of a homozygous genome results in fewer conflicting sites, leading to increased contiguity and reduced numbers of fragmented gene models, duplicate redundant contigs and incorrectly predicted gene paralogues^45^. For the genome asssembly of potato (*Solanum tuberosum*), a self-incompatible crop, the issue of heterozygosity was overcome using a homozygous doubled-monoploid derived through tissue culture^46,47^. In some cases, assembly of a heterozygous genome is unavoidable; for example, *Trifolium pratense* (red clover) is difficult to inbreed without severe loss of viability and vigour^48^. For *P*. *vulgaris*, we sampled a self-fertile long homostyle plant from an inbred population that originates from Wyke Champflower in Somerset, UK^31,32^. This highly homozygous individual allowed us to assemble the first *P*. *vulgaris* reference assembly of 411 Mb, which covers ~87% of the mean estimated 474 Mb genome^12,13^.

We previously identified the *P*. *vulgaris S* locus as the first complete structure of a heterostyly supergene; this region is hemizygous in thrum plants^27^. Here, our comparative analyses indicate that the hemizygous genetic architecture of the *S* locus is conserved in the *P*. *veris* thrum genome; furthermore, the five *S* locus genes identified in *P*. *vulgaris* are also present and show thrum-specific expression. Further analysis reveals reduced genomic read coverage across *S* locus coding sequence regions for *P*. *farinosa* (thrum), *P*. *forbesii* (thrum), and *P*. *scotica* (homostyle species) (Fig. 5), which would suggest the *S* locus is also absent from the pin chromosome in these species; however, due to phylogenetic distance this remains a hypothesis. Future mapping of genomic pin reads from distant species and PCR analysis across border sequences will confirm whether the *S* locus genetic architecture is conserved in the Primulaceae. *GLO*^*T*^^27^ and *CYP*^*T*^^28^ have nonetheless been identified as thrum-specific in a range of *Primula* species, which would support such a conclusion. Our previous estimate for the origin of the *S* locus using *GLO*^*T*^ and *GLO* sequences from six *Primula* species (including *P*. *farinosa*), is 51.7 MYA^27^. This age predates the divergence of the Primulaceae, and would be consistent with the apparent conservation of *S* locus genetic architecture, which would suggest a shared evolutionary history for heterostylous *Primula* species.

We annotated 24,599 genes in our *P*. *vulgaris* assembly. Our in-depth analysis of the *S* locus^27^ revealed *CCM*^*T*^ spans two contigs in the automated gene predictions; the gene number is therefore 24,598 if these two contigs are joined. The high percentage of RNA-Seq reads and CEGs mapping to the genome assembly suggests that most of the *P*. *vulgaris* genespace has been captured in the assembly. Further searches for degenerate TE sequences revealed 762 of the predicted *P*. *vulgaris* genes are potentially TE-related, which could further reduce the total to 23,836. Conversely, these genes may encode endogenous proteins, as is the case with the AP2 binding domain that is present in both plant developmental transcription factors (TFs) and integrases such as tn916^49^. Recruitment of TF binding domains from transposases or integrases is a potentially recurrent theme in evolution^49^, resulting in evolutionary mobile protein domains in different sequence contexts^50^. OrthoMCL analysis of *P*. *vulgaris* genes and their orthologues in four angiosperm species (Supplementary Fig. S3) reveals *GLO*^*T*^ as a paralogue of *GLO* in *P*. *vulgaris* as expected^27^, within a group containing MADS-box like genes in rice and tomato, as well as *PISTILATTA* from Arabidopsis. OrthoMCL did not identify paralogues for the remaining four *S* locus genes due to partial hits of low similarity, perhaps suggesting the duplication^27^ of *GLO* to *GLO*^*T*^ represents the most recent ancestral step in assembly of the *S* locus supergene; we note, however, that *CCM*^*T*^ does have sequence similarity (~90%) with another gene elsewhere in the genome^27^. Further investigation of the *P*. *vulgaris* genome to analyse gene families related to the *S* locus cluster will help to establish the ancestral steps leading to heterostyly.

*P*. *veris* and *P*. *vulgaris* are both heterostylous, closely-related, and can interbreed to produce hybrids known as “false oxlip”^51^. The two species have similarly-sized diploid genomes (*P*. *veris* = 479 Mb; *P*. *vulgaris* = 474 Mb)^13^, and might be expected to contain a similar number of genes. However, the reported *P*. *veris* genome assembly has 18,301 annotated genes^14^ (*P*. *vulgaris* = 24,599). To determine whether this represents a true difference in gene count, we compared coding sequences. We show a reduced percentage of mapped RNA-Seq reads and an absence of *P*. *vulgaris* genes in the *P*. *veris* genome and annotations. This difference could result from use of RNA-Seq datasets from a broader range of *P*. *vulgaris* tissues in the current study (Supplementary Table S2). For genes absent from the assembly, the highly-polymorphic *P*. *veris* read library, which combines genomic DNA from the heterozygous genomes of both a pin and a thrum plant^14^ would result in difficulties assembling the *P*. *veris* genome due to the presence of four haplotypes. The higher *P*. *veris* contig size cut-off (<888 bp)^14^ (vs. <200 bp for *P*. *vulgaris*) may also have removed true genomic content. In addition, the number of transcripts found in the reported *P*. *veris de novo* transcriptome assembly was much greater (25,409) than the number of predicted genes in the partial *P*. *veris* assembly^14^; although *de novo* transcriptomes often contain inflated numbers of transcripts^52^, this perhaps suggests there are more genes in the *P*. *veris* genome than present in the genome-guided gene predictions for this species^14^. These results suggest the different number of annotated genes is due to a greater percentage of genes captured in the *P*. *vulgaris* LH_v2 assembly and gene predictions, rather than inherent differences between the two species; ~24,000 genes is perhaps a reasonable estimate for the true gene number in these closely-related species.

Genes differentially expressed between pin and thrum flowers show over-representation of GO terms potentially relating to reproductive processes, and pathways that might affect cell wall modification in the development of dimorphic *P*. *vulgaris* flowers (Fig. 3c)^53^. Low expression for *S* locus genes other than *GLO*^*T*^ compared to the differentially expressed geneset (Supplementary Fig. S5) would be consistent with roles as master regulators that control groups of more highly expressed genes, through the modulation of phytohormones for example^27,28^, which are often produced in low concentrations^54^. Future detailed temporal analyses will determine *S* locus gene expression throughout flower development, and further our understanding of the dynamic action of these regulators on genome-wide gene expression. The *Primula S* locus was predicted to either contain, or be in close linkage with, genes encoding molecular specificities that determine SI^9^, based on the loss of SI in homostyle plants assumed to arise from recombination between dominant and recessive *S* alleles^4–6^. The *Primula S* (*Style length*) locus is distinct from the *S* loci defined in homomorphic self-incompatible species^11^; unlike homomorphic-SI *S* loci, the finding that the *Primula* thrum *S* locus haplotype has no counterpart in pin with which to recombine^27^, together with the observation that *GLO*^*T*^ and *CYP*^*T*^ mutations not only result in homostyle flowers, but also self-compatibility^27^, raises the possibility that the SI determinants associated with heterostyly might be present in the large differentially expressed geneset, under the control of *GLO*^*T*^ and *CYP*^*T*^, rather than located at the *S* locus itself. The existence of homozygous *P*. *vulgaris* homostyle plants such as the Somerset and Chiltern long homostyles, which are diploid for the *S* locus gene cluster^27^ (Fig. 5), highlights a further question to be resolved on the proposed recessive lethal gene linked to the *S* locus^55^, suggested as the basis for preventing homozygous thrums. These plants are the result of mutation, not recombination, which suggests SI is the key, very effective, mechanism that prevents homozygous (*S*/*S*) thrum plants from occurring^55^.

Our analyses reveal the number of genes with significantly higher expression in thrum is double the number with significantly higher expression in pin (Fig. 3a). The thrum-specific *S* locus alters pin floral architecture by reducing cell length in the style, increasing cell division below the point of anther attachment, modifying cell morphology in the upper corolla tube, and increasing pollen size^53^. These findings appear to suggest that the upregulation of a greater number of genes is required for development of a more complex set of thrum-specific traits. Refining these intricate developmental events in thrum most likely requires modulated expression of genes required in both pin and thrum flowers to bring about change from the default pin architecture, whilst maintaining a flower of the same overall size. Surprisingly, only eight genes with morph-specific expression (expressed uniquely in either pin or thrum) (Fig. 3b) are expressed at a significant level (including *GLO*^*T*^); there are no significantly enriched GO terms in this geneset (Supplementary Table S6). Genes controlled by the *S* locus could be required for default floral development, and are therefore present and expressed in both pin and thrum. These genes might be differentially expressed rather than morph-specific in expression due to the modification of floral architecture rather than generation of novel structure, enabling fine-scale tuning to maintain overall flower size in the two floral morphs.

*P*. *vulgaris* LH_v2 annotations generated in this study allowed us to investigate repeat sequences in the *S* locus region (Supplementary Fig. S4), with analyses revealing elevated TE sequence content (64%) compared to genome-wide (37%). This feature may be due to proximity of the *S* locus to the centromere^26^, a chromosomal region with characteristically high repeat content^56^. However, we reveal a remarkable contrast in the TE content of sequences immediately flanking the *S* locus (34%) in comparison to the *S* locus itself (64%), suggesting the elevated repeat content is not simply due to chromosomal location. TEs and insertional mutations can accumulate in regions with reduced recombination^57^, with a build-up of mutations potentially rendering a TE incapable of transposing. Since hemizygosity of the *S* locus in thrums precludes homologous recombination, the efficiency of selection would be reduced by restricting fixation of favourable allele combinations at linked loci^58^. The resulting build-up of deleterious mutations hitchhiking with beneficial mutations could lead to TE insertion without selective constraints, and larger introns; in some eukaryotes, a negative correlation of intron length with recombination rate has been observed^44^. We note the presence of large introns and intergenic gaps in the *S* locus region compared to both the flanking regions and genome-wide (Supplementary Fig. S4c). Reduced efficiency of selection as a result of decreased recombination rate has also been linked to short introns that are less than the minimum intron length required for the intron splicing reaction, defined as <80–90 bp in *Drosophila* studies^44^. However, the remaining introns, including those in *PUM*^*T*^, whilst noticeably smaller than the remarkably large (>5 kb) introns in *GLO*^*T*^ and *CYP*^*T*^, have sizes (median 426.5 bp) in line with the genome-wide average;  two of the *S* locus genes (*KFB*^*T*^, *CCM*^*T*^) have no introns.

The characterisation of the supergene controlling butterfly mimicry, as a single gene preserved in linkage disequilibrium by a chromosomal inversion^59^, is in stark contrast to the multiple gene *P*. *vulgaris S* locus region. Despite suppression of recombination, gene conversion events and rare multiple crossovers are still possible between standard and inverted chromosomes (inversion heterozygotes)^60,61^. For hemizygous *P*. *vulgaris* thrum plants, there is no opportunity for genetic exchange within the *S* locus supergene, due to the absence of the region from the pin chromosome. In hemizygous regions of the Y chromosome in humans, the presence of large, near-identical (>99.9% sequence identity) palindromic repeats that encompass the male-specific genes is well documented^62,63^. These repeats could be maintained by intra-chromosomal gene conversion, to facilitate the restoration of deleterious mutations by replacement with mutation-free gene copies^62,63^. However, for the *Primula S* locus, the *P*. *vulgaris* genome assembly reveals no near-identical repeated sequences or remarkably similar genes elsewhere in the genome.

Our findings suggest that the thrum-specific architecture of the *Primula* *S* locus is an evolutionary-conserved feature. In addition to functional analysis of the *S* locus genes and downstream pathways, this indicates that future investigations into the evolution of the region, including how its function is maintained given the above observations, will be the next big challenge to understand the *S* locus in Darwin’s primroses. The *P*. *vulgaris* genome assembly will, alongside the fully characterized *S* locus region, provide the resources to facilitate this research.

## Methods

### Genome assembly

SOAPdenovo v2.04^36^ was used to assemble contigs using genomic paired-end reads (Illumina HiSeq 2500) from an individual *P*. *vulgaris* long homostyle (Somerset) plant (-K 81); reads were generated as described previously^25,27^ (Supplementary Tables S1–S3). Contigs were scaffolded with paired-end reads, then mate-pair libraries, in order of ascending insert size (5, 7 and 9 kb; Supplementary Table S1) (−k 41); ≥5 links required to join contigs into a scaffold (pair_num_cutoff=5), contigs <100 bp excluded (-L 100). SOAP GapCloser was used to fill gaps in scaffolds (rd_len_cutof=70, map_len=35). BLASTN^64^ was used to identify and remove non-*Primula* contigs based on alignments to the NCBI “nr” database (≥90% identity; ≥50% coverage; alignment length ≥100 bp), and also additional *Primula* chloroplast sequences from *Primula sinensis* (KU321892.1), *Primula poissonii* (KF753634.1), and *Primula veris* (KX639823.1). Removal of contaminate contigs and contigs <200 bp produced the final assembly (LH_v2). To assess completeness and duplicated content in the assembly, *k*-mer hashes were generated for the paired-end reads and scaffolds using Jellyfish v2.2.0^65^, and compared with the K-mer Analysis Toolkit^34^. The proportion of *P*. *vulgaris* RNA-Seq reads, transcripts, and core eukaryotic genes mapping to the assembly was also evaluated (Supplementary Methods S1). Draft assembly of paired-end reads from an individual *P*. *veris* thrum plant (Supplementary Table S1) (VT_v1) was performed with ABySS v1.3.4 (*k* = 81)^66^; this assembly was not annotated.

### Genome annotation

RepeatModeler (open v1.0.7) (http://www.repeatmasker.org/RepeatModeler.html) was used to identify *de novo* repeat sequences in the LH_v2 assembly. These sequences were curated as described in Supplementary Methods S1. LH_v2 was annotated using the *de novo* repeat library with RepeatMasker (open v4.0.1; RMBlast v2.2.27) (http://www.repeatmasker.org/); additional classification of repeat elements was performed with TEclass v2.1.3^67^.

To generate a training set of genes for the eukaryotic gene-prediction software AUGUSTUS v2.7^68^, RNA-Seq reads from leaves, flowers, seed, seedlings, and roots (Supplementary Table S2) were aligned to LH_v2 with TopHat v2.0.11^69^. Transcripts from all libraries were assembled and merged using Cufflinks v2.1.1^70^, then filtered with full_lengther_next (https://rubygems.org/gems/full_lengther_next/) to identify full-length transcripts, and remove overlapping transcripts. AUGUSTUS was trained using 2,000 transcripts and the training parameters assessed with an additional 200 genes. In addition to *de novo* repeat annotations (“--nolow” and “--norna” RepeatMasker options applied), evidence sets based on protein, cDNA, and RNA-Seq intron and exon positions, were generated for gene prediction (Supplementary Methods S1).

Once trained, AUGUSTUS was used to identify genes in LH_v2 scaffolds. RNA-Seq reads from all libraries were aligned to scaffolds using AUGUSTUS predictions as “known transcripts” (-G parameter), then Cufflinks v2.1.1^70^ used to assemble transcripts. PASA2 April 25-2013 beta release^71^ was used to generate a modified annotation from these alignments; another round of alignment, assembly and correction was performed to generate final gene models. Functional annotation and identification of GO-terms and domains associated with the predicted genes was performed with AHRD^72^, Blast2GO^73^, and InterProScan5^74^ (Supplementary Methods S1).

### *Primula* gene comparison and expression

*P*. *vulgaris* and *P*. *veris*^14^ coding sequences (CDSs) were aligned against each other, and to the *P. veris*^14^ and *P. vulgaris* LH_v2 genome assemblies, using TBLASTX v2.2.31^64^. High Scoring Pairs (HSPs) with ≥95% sequence identity were extracted; total percentage coverage across each CDS was recorded, and the cumulative number of CDSs with each coverage plotted for each of the four alignments using R v3.2.0 (https://www.r-project.org/). OrthoMCL v2.0.9^75^ was used to find orthologous and paralogous gene groups based on all-vs-all alignments between proteins from *P*. *vulgaris*, *P*. *veris*, and other angiosperm species (Supplementary Methods S1).

RNA-Seq reads were generated in biological-replicate from 15–20 mm buds of four pin and four thrum *P*. *vulgaris* plants (siblings) (Supplementary Table S3), as described previously^27^. Differential expression analysis with Cufflinks v2.1.1 Cuffdiff ^76^, and GO term enrichment analysis with goatools (https://github.com/tanghaibao/goatools), was performed (Supplementary Methods S1).

*P*. *vulgaris S* locus genes^27^ were defined in the published *P*. *veris* GFF file of predicted genes^14^ (Supplementary Methods S1). Public RNA-Seq reads for *P*. *veris* pin and thrum flower RNA (BioProject PRJNA238546)^14^ (no replicates), and *P*. *veris* style and corolla tube RNA prepared from pin and thrum flowers (pooled from 25 plants)^28^ (BioProject PRJNA317964) were aligned to the published *P*. *veris* genome, prior to differential expression analysis (Supplementary Methods S1). PCR analysis of *S* locus genes was performed on cDNA from *P*. *veris* and *P*. *vulgaris* mixed-stage flower buds using the method described previously^27^; primers and amplification conditions listed in Supplementary Table S7.

### *Primula S* locus genomic read coverage

LH_v2 contigs forming the *S* locus region generated previously^27^ were removed from the *P*. *vulgaris* assembly and replaced with the contiguous 455,880 bp *S* locus and flanking sequences. BWA v0.7.12^77^ “aln” was used to map genomic reads from *P*. *veris* thrum, and *P*. *vulgaris* thrum and pin (Supplementary Table S1) to this assembly. SAMtools v0.1.19^78^ “depth” was used to compute read depth across the 455 kb region (Q30), which was normalized according to library size and plotted in 5 kb windows (Fig. 4a) with R v3.2.0 (https://www.r-project.org/). BWA v0.7.2 “mem”^79^ and Samtools v1.1.19^78^ were used for mapping and finding the depth of coverage of genomic reads from *Primula* species across predicted *P*. *vulgaris* LH_v2 *S* locus coding sequence positions (Fig. 5) (Supplementary Methods S1).

## Electronic supplementary material

Supplementary Information

## Data Availability

Sequencing data are available under BioProject PRJEB9683, PRJNA260472, PRJEB21011, GenBank accessions MF317487 to MF317489, and http://opendata.earlham.ac.uk/primula.
