# Supplementary material for: Primula vulgaris (primrose) genome assembly, annotation and gene expression, with comparative genomics on the heterostyly supergene
Source: Sci Rep. 2018 Dec 18;8:17942. doi: 10.1038/s41598-018-36304-4 (PMC6299000; doi:10.1038/s41598-018-36304-4)
Supplement: Supplementary file 1 — Supplementary Information [file 41598_2018_36304_MOESM1_ESM.pdf]

Supplementary Information

Article title: *Primula vulgaris* (primrose) genome assembly, annotation and gene expression, with comparative genomics on the heterostyly supergene

Authors: Jonathan M. Cocker, Jonathan Wright, Jinhong Li, David Swarbreck, Sarah Dyer, Mario Caccamo, and Philip M. Gilmartin

The following Supplementary Information is available for this article:

Figure S1 Frequency distribution (spectra) of *k*-mers in the *P. vulgaris* long homostyle assembly (LH_v2) and paired-end read library (LIB2558)

Figure S2 Contribution of genomic contigs to *Primula* genome assembly size

Figure S3 OrthoMCL analysis and *Primula* gene comparison

Figure S4 Transposable elements (TEs) and introns at the *Primula S* locus

Figure S5 Expression of *P. vulgaris S* locus genes compared to differentially expressed genes

Figure S6 RNA-Seq and PCR expression analysis of *S* locus genes in *P. veris*

Table S1 Illumina HiSeq2500 genomic paired-end read libraries used for assembly and sequence analyses

Table S2 Illumina HiSeq2000/2500 RNA-Seq paired-end read libraries used for gene predictions

Table S3 Illumina HiSeq2500 RNA-Seq paired-end read libraries used for differential expression analyses

Table S4 *Primula vulgaris* LH_v2 CEGMA analysis

Table S5 Classification of repeats in the *Primula vulgaris* LH_v2 genome

Table S6 GO term enrichment for genes with morph-specific expression

Table S7 Primers used in PCR expression analysis of *S* locus genes

Methods S1 Supplementary methods

Supplementary Figures S1-S6

Figure S1 Frequency distribution (spectra) of *k*-mers in the *P. vulgaris* long homostyle assembly (LH_v2) and paired-end read library (LIB2558)

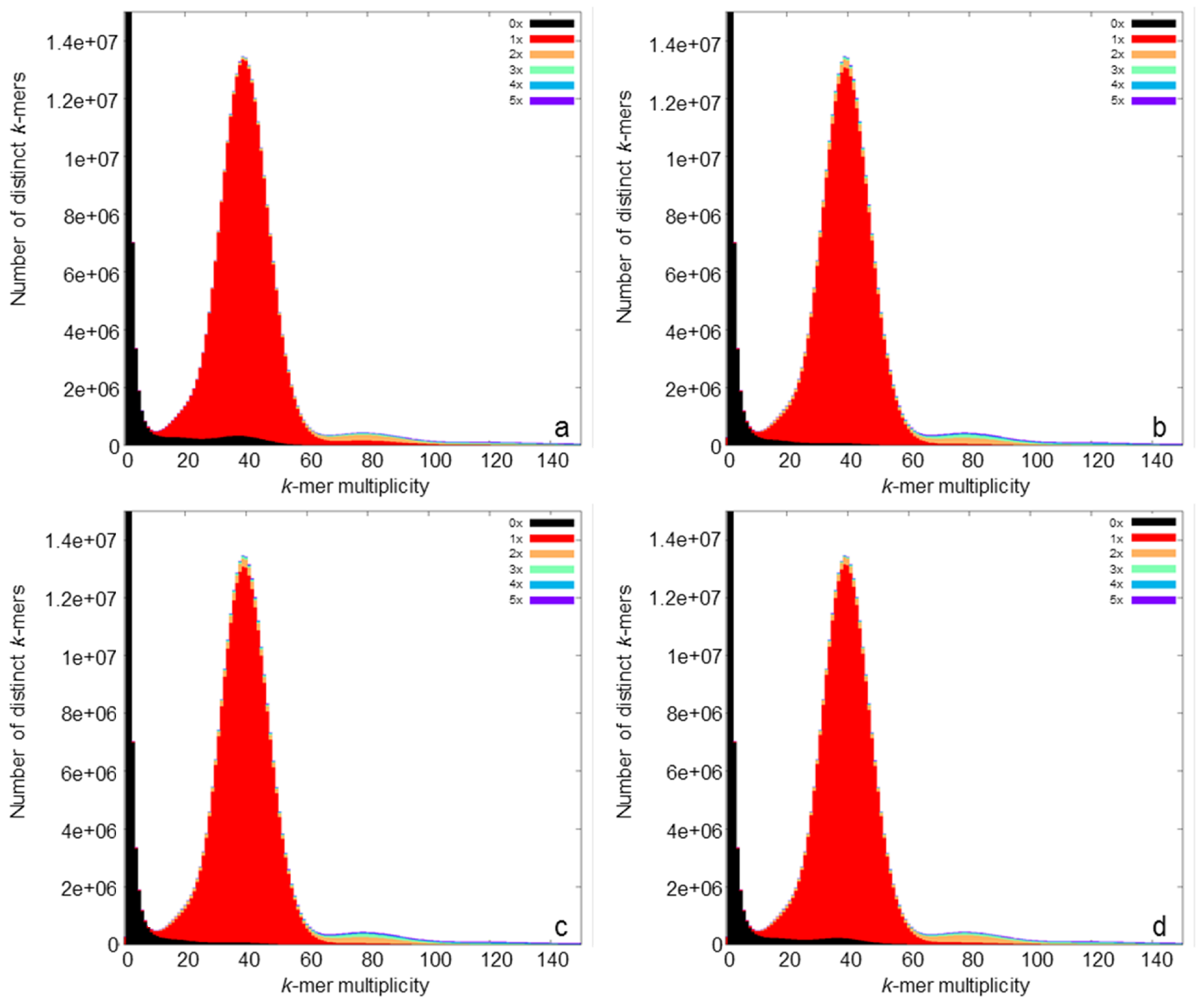

Frequency distribution (spectra) of *k*-mers (k=31) in the LIB2558 read library used for assembly of *P. vulgaris* LH_v2 (Table S1), and the copy number of these *k*-mers in the assembly at each stage: black = content absent from the assembly, red = content present once in the assembly, orange = twice, etc**.** **(a)** scaffolds before gap-filling, **(b)** after gap-filling, **(c)** contaminated contigs removed, **(d)** scaffolds less than 200 bp removed.

K-mer Analysis Toolkit[1] was used to compare *k*-mers present in the *P. vulgaris* long homostyle (LH_v2) assembly, and the LIB2558 read library from which it was generated (Fig. S1). We conclude that the assembly incorporates the majority of true genomic content that lies beyond the first local minima; *k*-mers in the exponential phase of the plot, that represent unique sequencing errors present in low frequencies[2] are, for the most part, excluded from the generated contigs (0x coverage in black). The homozygous content is generally present only once in the assembly (red); duplication is minimal, with *k*-mers from the main peak that do not appear in the assembly being small in number compared to *k*-mers that are included. The analysis suggests the assembly is highly "collapsed", with few alternate contigs that would otherwise result from heterozygous regions of the genome that cannot be resolved to consensus sequence[1,3]. Gap-filling reduced the number of read *k*-mers absent from the assembly (reduced black peak) and slightly increased *k*-mers found multiple times (Fig. S1 a to b). This step indicates a trade-off where extra sequence from the reads is being incorporated into the assembly by gap-filling, but some duplicated content is also introduced. There was no change to the observed *k*-mer spectra when contaminated contigs were removed (Fig. S1 b to c), and removing sequences < 200 bp in length did not remove a large portion of the genomic content from the assembly (Fig. S1c to d).

Figure S2 Contribution of genomic contigs to *Primula* genome assembly size

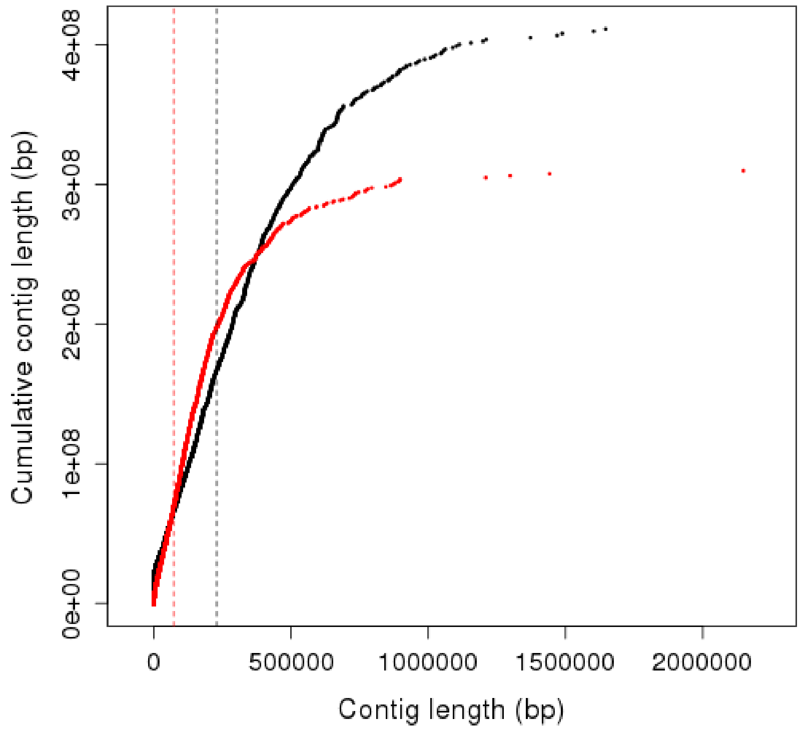

Contribution of genomic contigs of each length (x-axis) to the total cumulative contig length (y-axis) for *P. veris* (red)[4] and *P. vulgaris* LH_v2 (black) (current study). Dashed lines indicate the respective NG50 values for the assemblies: red = *P. veris* (73.3 kb), black = *P. vulgaris* (229.8 kb).

Figure S3 OrthoMCL analysis and *Primula* gene comparison

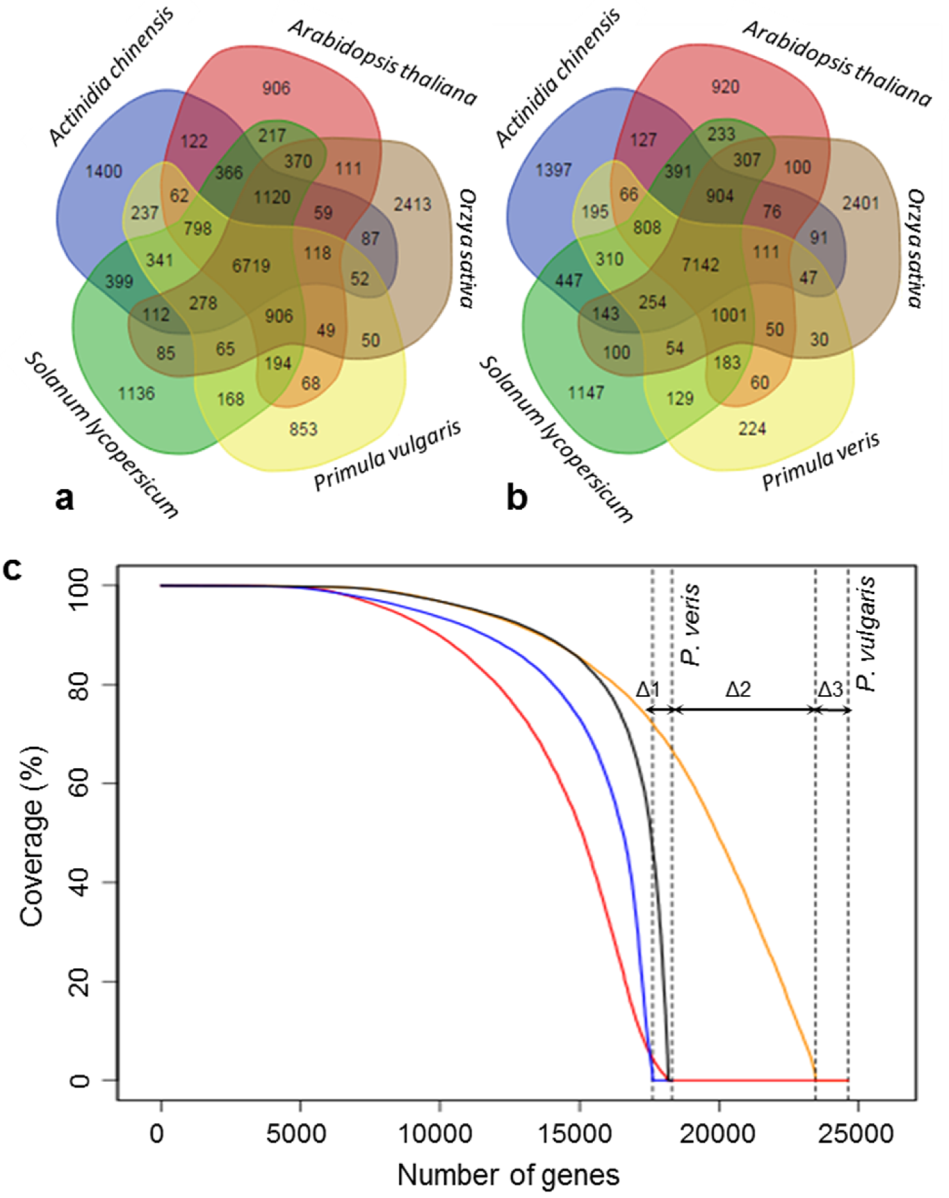

(a) OrthoMCL[5] analysis showing gene orthologues between *P. vulgaris* and four angiosperm species, with alignments based on predicted protein sequences in the LH_v2 genome; **(b)** corresponding OrthoMCL analysis using predicted protein sequences from a published *P. veris* genome assembly[4]; **(c)** comparison of genes annotated in *P. vulgaris* LH_v2 and published *P. veris* genome assemblies: total coverage of HSPs (High Scoring Pairs) with ≥ 95% sequence identity in TBLASTX alignments. LH_v2 coding sequences aligned to *P. veris* contigs

(orange), and *P. veris* coding sequences (red). *P. veris* coding sequences mapped to LH_v2 contigs (black), and LH_v2 coding sequences (blue). Dotted lines indicate total number of genes annotated in each genome (*P. veris* = 18,301, *P. vulgaris* = 24,599). Δ1 = number of *P. veris* coding sequences (n=685) with no coverage in LH_v2 coding sequences (17,616 of 18,301 present); Δ2 = number of LH_v2 coding sequences (n=6,501) with no coverage in *P. veris* coding sequences (18,098 of 24,599 present); Δ3 = number of LH_v2 coding sequences (n=1,166) with no coverage in *P. veris* genome assembly (23,433 of 24,599 present). *P. veris* genes (n=130) with no coverage in the *P. vulgaris* genome (18,171 of 18,301 present) is not indicated.

Supplementary Fig. S3c shows that 6,501 of 24,599 coding sequences (26.43%) in the *P. vulgaris* LH_v2 assembly have no coverage (> 95% identity) in 18,301 *P. veris* coding sequences (CDSs)[4]. The comparatively shallower slope for these alignments suggests reduced breadth of coverage for gene sequences that are present, supported by fewer CEGs with complete coverage in the *P. veris* genome (79.84%)[4], vs. 89.92% in *P. vulgaris* (Supplementary Table S4). From these 6,501 CDSs, 1,166 show no coverage in *P. veris* genomic contigs; alignment of RNA-Seq reads used for *P. veris* gene prediction is consistent with this, with mean overall and concordant pair alignment rates of 82.5% and 75.7%, compared to 91.1% and 85.0% for *P. vulgaris*. The reciprocal analysis reveals 685 *P. veris* CDSs absent from the *P. vulgaris* geneset, with 130 absent from *P. vulgaris* contigs. The reported number of CDSs in the published *P. veris* assembly[4] is 19,507, however, the available file contains 18,301 coding sequences; the ≥ 888 bp contig size cut-off in the final assembly probably removed some genes. TransposonPSI (http://transposonpsi.sourceforge.net/) reveals that up to 762 genes in *P. vulgaris*, and 226 genes in *P. veris*, potentially contain degenerate TEs, which may explain a subset of the 6,501 *P. vulgaris* coding sequences absent from *P. veris* annotations (see discussion). Further analysis of *P. vulgaris* genes was undertaken to identify homologues; alignments to angiosperm species and analysis with OrthoMCL[5] produced clusters of related proteins (Supplementary Fig. S3a). Predicted *P. veris* proteins[4] were also aligned to these species (Fig. S3b). For *P. vulgaris*, 19,861 orthologous gene groups were identified, which is comparable to 19,448 for *P. veris*; this might be expected for genes that are well-conserved, and therefore used as evidence in annotation pipelines[6]. The difference in the number of *P. vulgaris*-specific (853) and *P. veris*-specific (224) groups might be caused by missing *Primula*-

specific genes in *P. veris* affecting the clustering algorithm such that their homologues are recognized as more closely related to genes in other species.

Figure S4 Transposable elements (TEs) and introns at the *Primula S* locus

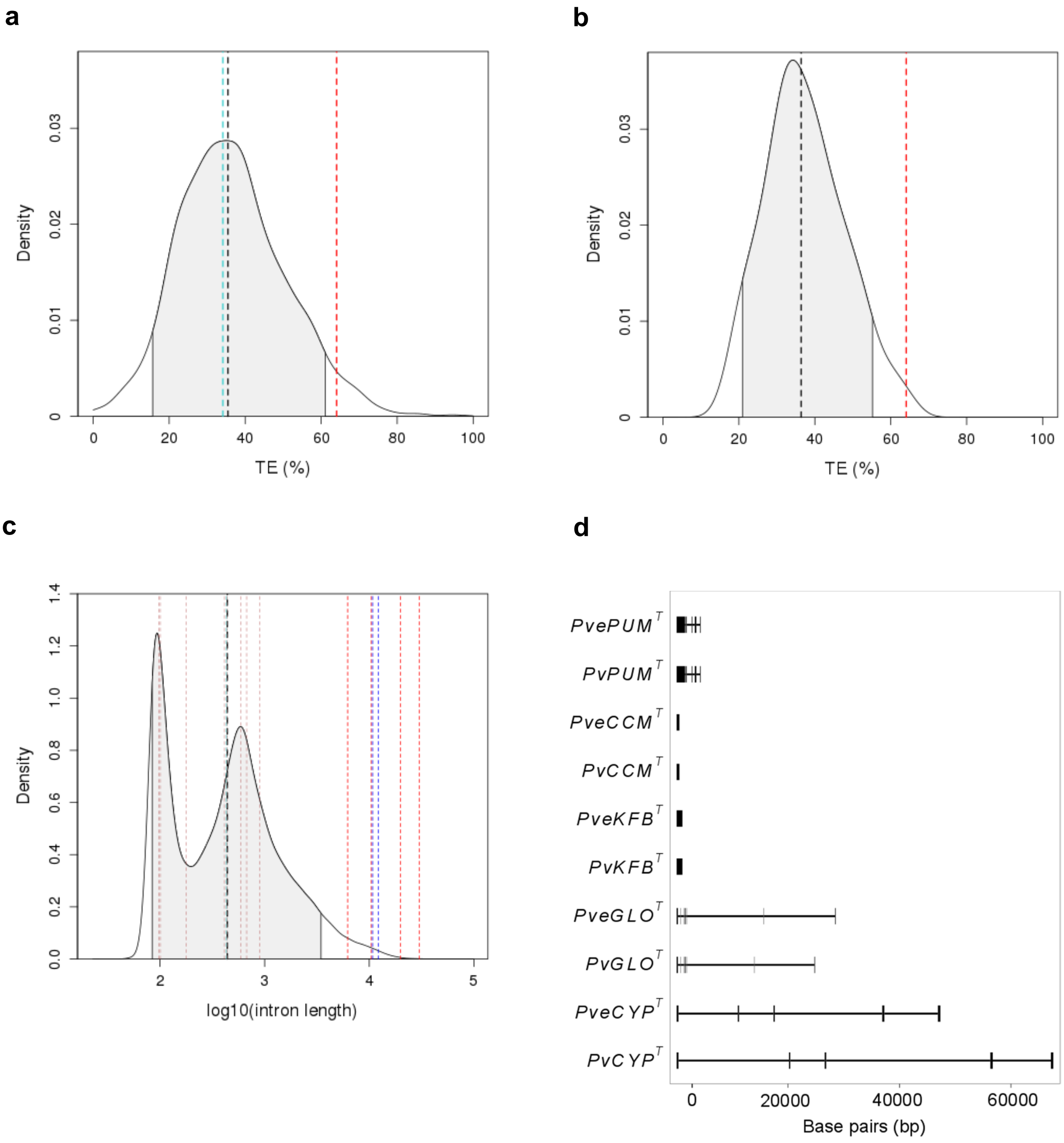

(a) Density plot of Transposable Element (TE) percentage (%) for contigs (> 10kb) in the *P. vulgaris* LH_v2 genome assembly (n=2,409); dashed black line = median (35.47%); dashed red line = TE percentage of the *P. vulgaris S* locus (64.07%); dashed teal line = TE percentage (34.67%) of concatenated left and right *P. vulgaris S* locus flanking regions (171 kb); shaded area (grey) = 95% quantile; **(b)** as in (a) for contigs ±20% the length (278 kb) of the *P. vulgaris S* locus (n=218); median (dashed black line) = 36.39%; **(c)** density plot of $\log_{10}$-transformed lengths (bp) of *P. vulgaris* introns (n=133,334); dashed lines = median $\log_{10}$(intron length bp)

(black), median $\log_{10}$(intron length bp) of regions flanking the *S* locus (teal), $\log_{10}$(length bp) of *P. vulgaris S* locus introns < 5 kb (brown), and $\log_{10}$(length bp) of *P. vulgaris S* locus introns > 5 kb: *GLO*^{T} (blue) (12172 bp, 10755 bp), *CYP*^{T} (red) (19870 bp, 6205 bp, 29971 bp, 10469 bp); **(d)** *P. veris* (*Pve*) and *P. vulgaris* (*Pv*) *S* locus gene models; vertical lines = exons, horizontal lines = introns, fully expanded intron sizes are displayed; bp = base pairs.

Figure S5 Expression of *P. vulgaris S* locus genes compared to significantly differentially expressed genes

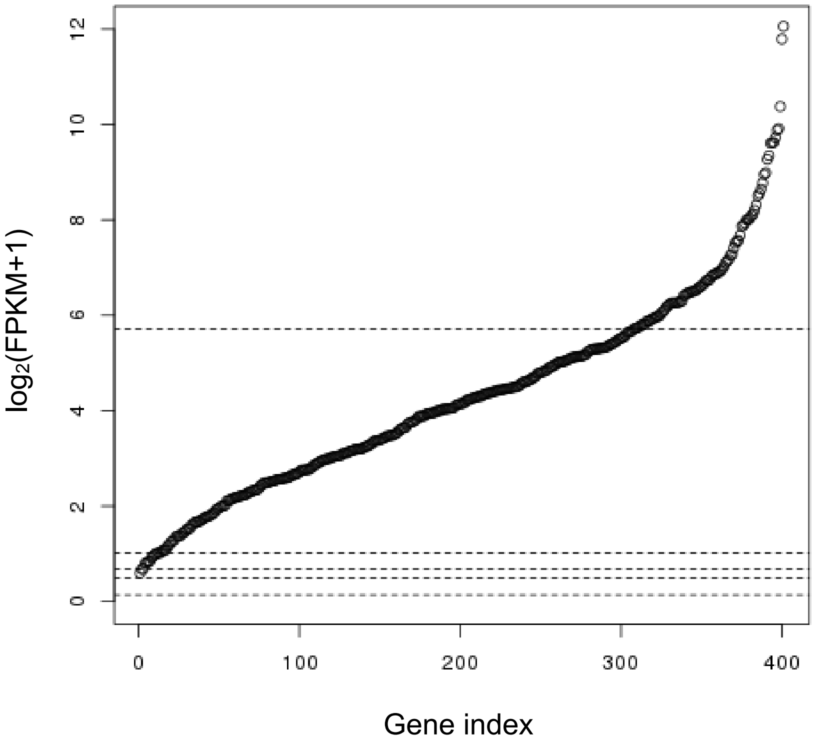

Log_2(FPKM+1) expression of genes significantly differentially expressed between pin and thrum flowers (401) (FDR < 0.05) (FPKM = Fragments Per Kilobase of transcript per Million fragments mapped, FDR = False Discovery Rate). Expression is shown for either pin or thrum flowers, whichever is higher. Dotted lines indicate log_2(FPKM+1) expression levels in thrum for the five *S* locus genes[7], from top to bottom: *GLO*^{T}, *CYP*^{T}, *PUM*^{T}, *KFB*^{T}, *CCM*^{T}.

Figure S6 RNA-Seq and PCR expression analysis of *S* locus genes in *P. veris*

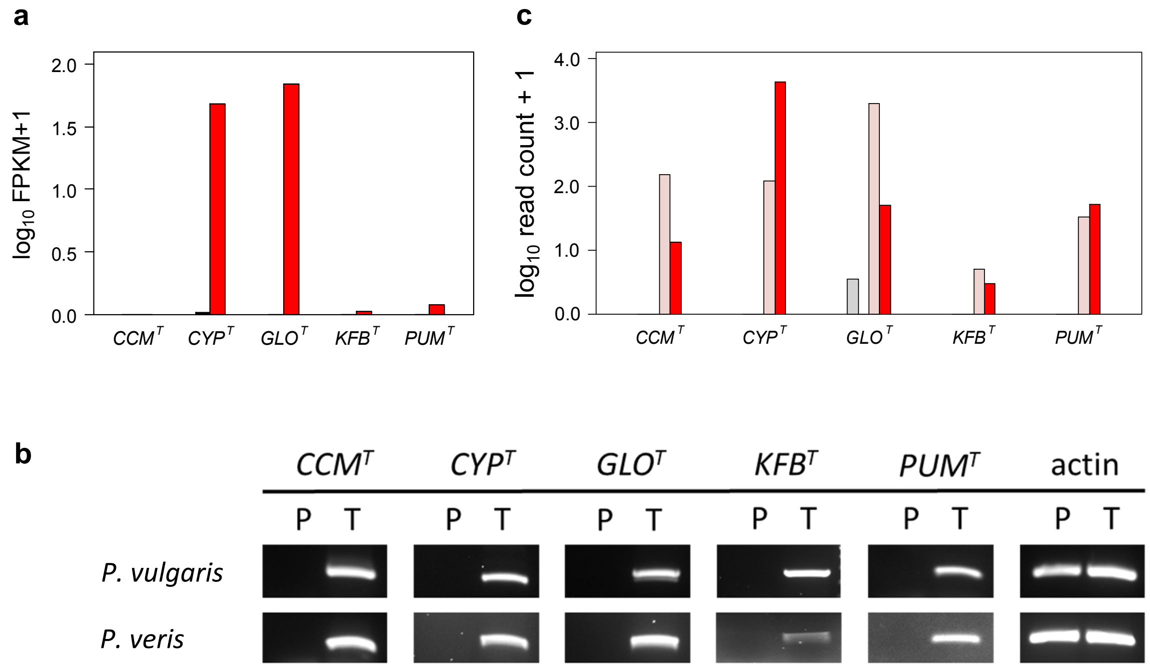

(a) RNA-Seq expression $\log_{10}$(FPKM+1) of *P. veris* pin (black) and thrum (red) flower RNA (unreplicated)[4] for the five *S* locus genes identified in *P. vulgaris*[7]; **(b)** RNA-Seq expression $\log_{10}$(DESeq2 normalised read count + 1) of *P. veris* RNA from styles and corolla tubes of pin and thrum flowers (pooled from 25 plants)[8] (three replicates) for the five *S* locus genes: black = pin corolla tube, grey = pin style, salmon = thrum corolla tube, red = thrum style; **(c)** PCR analysis of cDNA showing presence or absence of expression for the *S* locus genes (actin as a control) using RNA from *P. veris* and *P. vulgaris* pin (P) and thrum (T) mixed-stage flower buds; sourced and cropped from multiple gels.

Supplementary Tables S1-S7

Table S1 Illumina HiSeq2500 genomic paired-end read libraries used for assembly and sequence analyses

Library	Material	Type	Insert size (bp)	Read count
LIB2558	Long homostyle (S)	Genomic	522	140114901
LIB5215	Long homostyle (S)	Genomic	4131	40450009
LIB5216	Long homostyle (S)	Genomic	6675	54234033
LIB5217	Long homostyle (S)	Genomic	8818	53977718
LIB1732	Pin parent	Genomic	423	205079089
LIB1167	Thrum parent	Genomic	368	147913358
LIB3564	*P. veris* thrum*	Genomic	556	179871647
LIB4658	Long homostyle (C)*	Genomic	562	92463870
LIB6101	*P. scotica* (h)*	Genomic	430	147344074
LIB6102	*P. farinosa* thrum*	Genomic	433	142115678

Libraries from *Primula vulgaris* unless stated. Long homostyle sequencing reads are derived from plants originating from two UK populations[9]: S = Somerset (UK), C = Chilterns (UK). Long homostyle (S) reads were used for the *P. vulgaris* LH_v2 assembly; h = homostyle species. Libraries correspond to those in Supplementary Table S1a of our previous *S* locus studies[7] (PRJEB9683) unless indicated by "*" (PRJEB21011). Read count refers to the number of paired-end reads.

Table S2 Illumina HiSeq2000/2500 RNA-Seq paired-end read libraries used for gene predictions

Library	Material	Type	Insert size (bp)	Read count
LIB668	Pin mixed-stage flower	RNA	223	100768798
LIB669	Thrum mixed-stage flower	RNA	180	81045610
LIB976	*Oakleaf* open flower	RNA	220	24995179
LIB977	Pin open flower	RNA	209	33400153
LIB978	*Oakleaf* leaf	RNA	219	14310589
LIB979	Pin leaf	RNA	215	45723021
LIB4734	Thrum flower	RNA	214	75716502
LIB4735	Mixed root	RNA	199	77754060
LIB4736	Fresh seed	RNA	205	63843125
LIB4737	Seedlings	RNA	235	80545495

Libraries derived from *Primula vulgaris*. LIB668-LIB669 and LIB976-979 sequenced on Illumina HiSeq2000 platform. LIB4734-LIB4737 sequenced on Illumina HiSeq2500 platform. Libraries correspond to those in Supplementary Table S1a of our previous *S* locus studies[7] (PRJNA260472 and PRJEB9683). Read count refers to the number of paired-end reads.

Table S3 Illumina HiSeq2500 RNA-Seq paired-end read libraries used for differential expression analyses

Library	Material	Type	Insert size (bp)	Read count
LIB8234	Pin flower (1)	RNA	296	39027602
LIB8235	Pin flower (2)	RNA	278	26497129
LIB8236	Pin flower (3)	RNA	318	31792278
LIB8237	Pin flower (4)	RNA	284	34838987
LIB8238	Thrum flower (1)	RNA	296	30318958
LIB8239	Thrum flower (2)	RNA	295	32575413
LIB8240	Thrum flower (3)	RNA	269	23971121
LIB8241	Thrum flower (4)	RNA	286	38550763

Libraries derived from *Primula vulgaris*. Number in brackets indicates biological replicate number for RNA-Seq data. Libraries correspond to those in Supplementary Table S1a of our previous *S* locus studies[7] (PRJNA260472 and PRJEB9683). Read count refers to the number of paired-end reads.

Table S4 *Primula vulgaris* LH_v2 CEGMA analysis

Core Eukaryotic Genes	No. proteins	Completeness (%)
Complete representation	223	89.92
Group 1	61	92.42
Group 2	46	82.14
Group 3	53	86.89
Group 4	63	96.92
Partial representation	241	97.18
Group 1	63	95.45
Group 2	53	94.64
Group 3	61	100.00
Group 4	64	98.46

The number and percentage of 248 ultra-conserved Core Eukaryotic Genes (CEGs) present (either complete or partial representation) in the predicted geneset of *Primula vulgaris* LH_v2, as determined by CEGMA (v2.5)[10].

Table S5 Classification of repeats in the *Primula vulgaris* LH_v2 genome

**Repeat type**	**Length (bp)**	**% in genome**	**% in repeat**
**Interspersed repeats**	153945885 (153945885)	37.44 (37.44)	96.15 (96.15)
**Class I: Retroelement**	62615656 (95912158)	15.23 (23.33)	39.11 (63.68)
**LTR Retrotransposon**	54323780 (66959990)	13.21 (16.28)	33.93 (41.82)
Copia	39875915 (39875915)	9.70 (9.70)	24.91 (24.91)
Gypsy	12544567 (12544567)	3.05 (3.05)	7.83 (7.83)
Other LTR	1903298 (14539508)	0.46 (3.54)	1.19 (9.08)
**non-LTR Retrotransposon**	8291876 (28952168)	2.02 (7.04)	5.18 (18.08)
SINE	1085346 (1363804)	0.26 (0.33)	0.68 (0.85)
LINE	7206311 (20717729)	1.75 (5.04)	4.50 (12.94)
other non-LTR	219 (6870635)	< 0.01 (1.67)	< 0.01 (4.29)
**Other Class I**	219 (6045423)	< 0.01 (1.47)	< 0.01 (3.78)
**Class II: DNA transposon**	19522480 (52043722)	4.75 (12.66)	12.19 (32.50)
**Unclassified interspersed**	71807749 (5990005)	17.46 (1.46)	44.85 (3.74)
**Other**	6164398 (6164398)	1.50 (1.50)	3.85 (3.85)
**Total uncorrected**	160110283 (160110283)	38.94	100.00 (100.00)
**Total corrected**	152243370	37.03	

Repeat sequences annotated in the 411.1 Mb *Primula vulgaris* LH_v2 genome assembly, using RepeatMasker with a *de novo* repeat library generated for *P. vulgaris*. Length (base-pairs) and percentage (%) in the genome and repetitive portion of the genome is shown for each repeat class (uncorrected) (default RepeatMasker options); additional classification with TEclass[11] is shown in brackets. Total uncorrected = total length (bp) of all repeat types based on parsed RepeatMasker ".out" output, without correction for overlaps and fragmented annotations (default RepeatMasker options). Total corrected = total length (bp) of all repeat types based on RepeatMasker ".tbl" output, corrected for overlaps and fragmented annotations ("--nolow" and "--norna" RepeatMasker options applied). Totals shown are based on LH_v2 prior to removal of contigs containing *Primula* chloroplast sequences.

Table S6 GO term enrichment for genes with morph-specific expression

GO ID	Description	Ratio in study	Ratio in pop	P-value uncorrected	FDR
GO:0005488	binding	38/993	507/24599	0.000337	0.172
GO:0016818	hydrolase activity, acting on acid anhydrides, in phosphorus-containing anhydrides	4/993	11/24599	0.000694	0.298
GO:0012511	monolayer-surrounded lipid storage body	3/993	7/24599	0.00203	0.778
GO:0006974	cellular response to DNA damage stimulus	2/993	4/24599	0.00925	1
GO:0071840	cellular component organization or biogenesis	2/993	4/24599	0.00925	1
GO:0043170	macromolecule metabolic process	5/993	35/24599	0.0126	1
GO:0015074	DNA integration	9/993	94/24599	0.0137	1
GO:0004506	squalene monooxygenase activity	2/993	5/24599	0.015	1
GO:0006259	DNA metabolic process	6/993	54/24599	0.0212	1
GO:0005576	extracellular region	8/993	88/24599	0.0257	1
GO:0010205	photoinhibition	2/993	8/24599	0.0388	1
GO:0015149	hexose transmembrane transporter activity	1/993	1/24599	0.0404	1
GO:0005976	polysaccharide metabolic process	1/993	1/24599	0.0404	1
GO:0003994	aconitate hydratase activity	1/993	1/24599	0.0404	1
GO:0015146	pentose transmembrane transporter activity	1/993	1/24599	0.0404	1
GO:0015166	polyol transmembrane transporter activity	1/993	1/24599	0.0404	1
GO:0015750	pentose transport	1/993	1/24599	0.0404	1
GO:0010262	somatic embryogenesis	1/993	1/24599	0.0404	1

GO term enrichment analysis for genes specifically expressed in either pin or thrum (Fig 3b); no GO terms are significantly overrepresented in this geneset (FDR ≥ 0.1).

Table S7 Primers used in PCR expression analysis of *S* locus genes

**Primer**	**Sequence 5' – 3'**	**Amplification conditions**
CCMTc-F	CCGTATTTCCGAAACTCACATTC	95°C 3 min; 95°C 30 sec, 60°C 30 sec, 72°C 1 min, x35 cycles; 72°C for 5 min.
CCMTc-R	GCTAGCACAAGGGTAAGTGAAC	As above
GLOTc-F	GAGAACAAGAAAGCTAGAGAG	95°C 3 min; 95°C 30 sec, 58°C 30 sec, 72°C 1 min, x35 cycles; 72°C for 5 min.
GLOTc-R	CTCAAGGTTGATATTTCAGGTT	As above
CYPTc-F	CGTGGACCACTGTTCTCCTT	95°C 3 min; 95°C 30 sec, 61°C 30 sec, 72°C 1 min, x35 cycles; 72°C for 5 min
CYPTc-R	GAACTCGGTTGCTTCCTGAC	As above
PUMTc-F	AGCCCACATGTTCTTGGTTC	95°C 3 min; 95°C 30 sec, 61°C 30 sec, 72°C 1 min, x35 cycles; 72°C for 5 min.
PUMTc-R	GCCTCAACCAAACGTTGACT	As above
PvKFBTc-F	CCACCCAGTTAAGGACGTG	95°C 3 min; 95°C 30 sec, 60°C 30 sec, 72°C 1 min, x35 cycles.
PvKFBTc-R	GTTTGCCCTTCACCAACAT	As above
PveKFBTc-F	CCCTCCCACTTCGTGAATAA	95 °C 3 min; 95 °C, 30 sec, 58 °C, 30 sec, 72°C 1 min for 5 cycles, then 95°C 30 sec, 55°C 30 sec, 72 °C 1 min x33 cycles; then 72 °C for 5 min.
PveKFBTc-R	CTCCCGCGACGTATACATGCC	As above

Primers used in PCR expression analysis of the *Primula S* locus genes (Supplementary Figure S6).

Methods S1 Supplementary methods

Assembly validation

Jellyfish v2.2.0[12] was used to generate *k*-mer hashes (-m 31) for thrum, pin and long homostyle paired-end read libraries (Supplementary Table S1), with output from the "histo" function plotted in R v3.2.0 (https://www.r-project.org/) (Fig. 2); hashes were also generated for LH_v2 scaffolds at each stage of the assembly (-m 31) (see Supplementary Fig. S1). *K*-mer Analysis Toolkit (KAT)[1] "comp" was used to compare *k*-mer hashes for the paired-end reads and scaffolds to evaluate completeness and duplicated content at each stage of the assembly, and *k*-mer spectra plotted with the KAT 'plot' function (Supplementary Fig. S1). To analyse the proprtion of RNA-Seq reads mapping to the assembly, RNA-Seq reads (Supplementary Table S2) were filtered for ribosomal RNA with sortmeRNA v1.9[13] and quality-trimmed (Q20) by trim galore v0.3.3 (http://www.bioinformatics.babraham.ac.uk/projects/trim_galore/), prior to short-read alignment with TopHat v2.0.11[14]. CEGMA v2.5[10] was used to evaluate the presence of a set of 248 Core Eukaryotic Genes (CEGs) in the LH_v2 genome assembly (intron_size=50000). *P. vulgaris KNOTTED*-like and B-function MADS-box genes[15-17] were also mapped to the *P. vulgaris* geneset with Exonerate v2.2.0[18] to confirm the presence and accuracy of the gene model predictions.

Repeat annotation

RepeatModeler (open v1.0.7) (http://www.repeatmasker.org/RepeatModeler.html) was used to identify *de novo* repeat sequences in the LH_v2 assembly. These sequences were aligned to Pfam-A (curated thresholds) and Pfam-B (e-value $1x10^{-4}$)[19] with HMMer v3.1b1 "hmmscan"[20]; those with no alignments, or alignments to transposable element (TE) domains, were retained; domains were considered TE-related based on alignments with HMMer v3.1b1 "hmmscan" (e-value $1x10^{-4}$) to the library of TEs included in the RepeatRunner package (http://www.yandell-lab.org/software/repeatrunner.html). For sequences identifying both TE and non-TE domains, those with no BLASTX v2.2.28[21] hits to the NCBI "nr" database (e-value $1x10^{-4}$), or classified as transposition-associated based on manual review, were retained; the remainder were discarded. The LH_v2 assembly was annotated using the *de novo* repeat library with RepeatMasker (open v4.0.1; RMBlast v2.2.27; TRF v4.04) (http://www.repeatmasker.org/); additional classification of repeat elements was performed with TEclass v2.1.3[11].

Evidence sets for gene prediction

The following evidence sets were generated for gene prediction with AUGUSTUS v2.7[22]: (i) Protein alignments: amino acid sequences were obtained for *Solanum lycopersicum* v2.4, *Solanum tuberosum* v3.4, *Mimulus guttatus* v2.0, *Capsicum annuum* v2.0, *Actinidia chinensis* (NA), *Nicotiana benthemiana* v0.4.4, and additional asterid species in the NCBI protein database (http://www.ncbi.nlm.nih.gov/). The sequences were masked for low-complexity regions using BLAST v2.2.28[21] and aligned to LH_v2 with Exonerate v2.2.0[18] (--model "protein2genome"); the alignments were filtered to retain those with minimum 90% sequence identity and 70% coverage, then converted to GFF (General Feature Format). (ii) *Primula* cDNA: transcript assemblies generated from 454 sequencing of pin and thrum RNA were softmasked (dustmasker), then aligned to the repeatmasked LH_v2 scaffolds with GMAP[23]. The output was filtered for > 90% identity alignments, before conversion to GFF. (iii) RNA-Seq introns: the junctions.bed files from the Tophat alignment (see Methods) were converted to GFF, then merged and filtered based on the number of reads per junction. (iv) RNA-Seq exons: the TopHat BAM files were converted to WIG files, then to GFF.

Functional annotation

BLASTP v2.2.22[24] (e-value $1x10^{-4}$) was used to align protein sequences (principal isoforms) from predicted LH_v2 genes to proteins from Uniprot/trEMBL, Uniprot/Swissprot (http://www.uniprot.org/), and TAIR10 (https://www.arabidopsis.org/). Functional annotation was performed with AHRD (Automated assignment of Human Readable Descriptions) (https://github.com/groupschoof/AHRD)[25] (weighting applied; TAIR = 50, trEMBL = 10, Swissprot = 100). GO (gene ontology) terms were assigned with Blast2GO[26] using BLASTX v2.2.28[21] searches against the NCBI "nr" database (e-value $1x10^{-4}$); domains were annotated with InterProScan5[27]. TransposonPSI (http://transposonpsi.sourceforge.net/) was used to detect additional degenerative repetitive elements in coding sequences from *P. veris*[4] and *P. vulgaris* LH_v2.

RNA-Seq differential expression

RNA-Seq reads (Illumina HiSeq2500) were generated using 15-20 mm *P. vulgaris* floral buds from four pin plants and four thrum plants (siblings from a controlled cross) (Supplementary Table S3); generated as described previously[7]. Reads were screened for ribosomal RNA using sortmeRNA v1.9[13], and quality-trimmed with trim galore v0.3.3 (Q20) (http://www.bioinformatics.babraham.ac.uk/projects/trim_galore), before alignment to LH_v2

with TopHat v2.0.13, and assembly with Cufflinks v2.2.1[28] using LH_v2 gene models as a guide. Cuffdiff (Cufflinks v2.2.1)[28] was used to find genes differentially expressed between pin and thrum flowers (FDR < 0.05), and genes expressed in only one morph. R v3.2.0 (https://www.r-project.org/) was used to plot log_2(fold change) in FPKM for differentially expressed genes, and log_{10} difference in FPKM+1 for genes with morph-specific expression. GO term overrepresentation in these genesets compared to GO terms attached to LH_v2 genes (in functional annotations) was determined using the goatools package for Python (https://github.com/tanghaibao/goatools) (FDR < 0.1).

***P. vulgaris* and *P. veris* gene comparison**

TBLASTX v2.2.31+[21] was used to align *P. veris* coding sequences (CDSs)[4] to *P. vulgaris* LH_v2 CDSs and genome contigs, and LH_v2 CDSs to published *P. veris* genomic contigs and CDSs. For genes with multiple predicted isoforms, the isoform with suffix ".1" was regarded as the principal isoform and used in this analysis. High Scoring Pairs (HSPs) with ≥ 95% sequence identity were extracted; the total percentage coverage across each CDS was recorded, and the cumulative number of CDSs with each coverage plotted for the four alignments using R v3.2.0 (https://www.r-project.org/).

Orthologous gene groups

Identification of orthologous and paralogous gene groups with OrthoMCL v2.0.9[5] was performed as described in the OrthoMCL user guide (inflation factor=1.5) using protein sequences from (i) *P. vulgaris* LH_v2, and (ii) *P. veris*[4]. BLASTP v2.2.28+[21] was used to perform all-vs-all alignment of these proteins (e-value=1x10-5; "seg" and "soft masking" options applied) with protein sequences from *Actinidia chinensis* (http://bioinfo.bti.cornell.edu/cgi-bin/kiwi/home.cgi), *Orzya sativa* (version 7, http://rice.plantbiology.msu.edu), *Arabidopsis thaliana* (TAIR10, https://www.arabidopsis.org) and *Solanum lycopersicum* (version 2.4, http://solgenomics.net/organism/Solanum_lycopersicum/genome); only principal isoforms were used.

***Primula veris S* locus gene expression**

The GFF file of predicted genes in the published *P. veris* genome assembly[4] was curated to correct (*GLO^T*, *CYP^T*, *KFB^T*) or add (*PUM^T*, *CCM^T*) orthologues of the *P. vulgaris S* locus gene models[7] based on alignments with Exonerate v2.2.0[18] to the published *P. veris* genome

assembly[4]. GFF coordinates were used to plot *P. veris* gene structures using R v3.2.0 (https://www.r-project.org/) with introns > 1 kb plotted as 1 kb (Fig. 4b), and *P. veris* and *P. vulgaris* gene structures with intron lengths fully expanded (Supplementary Fig. S4d).

RNA-Seq reads for *P. veris* pin and thrum flower RNA (BioProject PRJNA238546)[4] (single RNA-Seq library for each floral form) were aligned to the published *P. veris* genome assembly[4] with TopHat v2.0.11[14] and assembled with Cufflinks v2.1.1[28] guided by our curated GFF file of predicted *P. veris* genes. Differential expression was carried out using Cuffdiff (Cufflinks v2.2.1)[29], and expression $\log_{10}$(FPKM+1) plotted for pin and thrum flowers with R v3.2.0 (https://www.r-project.org/).

RNA-Seq libraries for *P. veris* style and corolla tube RNA prepared from pin and thrum flowers (tissue pooled from 25 plants per library) (BioProject PRJNA317964)[8] were aligned to the published *P. veris* genome using HISAT v2.0.5[30], with exons and splice sites specified for the index build based on our curated GFF file of predicted *P. veris* genes. Quantification was performed with Stringtie v1.3.0[31] using the above GFF file as a guide; a matrix of read counts was then generated with prepDE.py and used in DESeq2[32] to produce normalized read counts that were plotted with R v3.2.0 (https://www.r-project.org/).

Primula S **locus genomic read coverage**

BWA v0.7.12[33] "mem" was used (default settings) to map genomic reads from *P. vulgaris*, *P. veris*, *P. farinosa*, *P. scotica* (Supplementary Table S1) and *P. forbesii* (PRJNA317964)[8] to the LH_v2 assembly incorporating the 455 kb *S* locus and flanking regions[7]. *P. vulgaris S* locus coding sequences[7] were aligned to the 455 kb *S* locus using Exonerate v2.2.0[18] to determine coding sequence positions. Read depth across positions defined by the coding sequences was determined with Samtools v0.1.19[34] (Q30), and normalized according to library size; depth was capped at 150 for *P. vulgaris* and *P. veris*, 100 for *P. farinosa* and *P. scotica*, and 50 *P. forbesii*. R v3.2.0 was used to plot read depth across the *S* locus coding sequence regions in 1 kb windows (step size=1) (Fig. 5) (each window considers the depths 500 bp to the left and right of centre); displayed median read depth is based on normalized counts with no depth cutoff.

References

1 Mapleson, D., Garcia Accinelli, G., Kettleborough, G., Wright, J. & Clavijo, B. J. KAT: a *K*-mer analysis toolkit to quality control NGS datasets and genome assemblies. *Bioinformatics* **33**, 574-576 (2017).

2 Sato, S. *et al.* The tomato genome sequence provides insights into fleshy fruit evolution. *Nature* **485**, 635-641 (2012).

3 Pryszcz, L. P. & Gabaldón, T. Redundans: an assembly pipeline for highly heterozygous genomes. *Nucleic Acids Research* **44**, e113 (2016).

4 Nowak, M. D. *et al.* The draft genome of *Primula veris* yields insight into the molecular basis of heterostyly. *Genome biology* **16**, 16 (2015).

5 Li, L. OrthoMCL: Identification of ortholog groups for eukaryotic genomes. *Genome Research* **13**, 2178-2189 (2003).

6 Holt, C. & Yandell, M. MAKER2: an annotation pipeline and genome-database management tool for second-generation genome projects. *BMC Bioinformatics* **12**, 1-14 (2011).

7 Li, J. *et al.* Genetic architecture and evolution of the *S* locus supergene in *Primula vulgaris*. *Nature Plants* **2**, 16188 (2016).

8 Huu, C. N. *et al.* Presence versus absence of *CYP734A50* underlies the style-length dimorphism in primroses. *eLife* **5**, e17956 (2016).

9 Crosby, J. L. Selection of an unfavourable gene complex. *Evolutionary Ecology Research* **3**, 212-230 (1949).

10 Parra, G., Bradnam, K., Ning, Z., Keane, T. & Korf, I. Assessing the gene space in draft genomes. *Nucleic Acids Research* **37**, 289-297 (2009).

11 Abrusan, G., Grundmann, N., DeMester, L. & Makalowski, W. TEclass—a tool for automated classification of unknown eukaryotic transposable elements. *Bioinformatics* **25**, 1329-1330 (2009).

12 Marçais, G. & Kingsford, C. A fast, lock-free approach for efficient parallel counting of occurrences of k-mers. *Bioinformatics* **27**, 764-770 (2011).

13 Kopylova, E., Noe, L. & Touzet, H. SortMeRNA: fast and accurate filtering of ribosomal RNAs in metatranscriptomic data. *Bioinformatics* **28**, 3211-3217 (2012).

14 Kim, D. *et al.* TopHat2: accurate alignment of transcriptomes in the presence of insertions, deletions and gene fusions. *Genome biology* **14**, 1-13 (2013).

15 Li, J. *et al.* The *S* locus-linked *Primula* homeotic mutant *sepaloid* shows characteristics of a B-function mutant but does not result from mutation in a B-function gene. *Plant Journal* **56**, 1-12 (2008).

16 Li, J. *et al. Hose in Hose,* an *S* locus-linked mutant of *Primula vulgaris* is caused by an unstable mutation at the *Globosa* locus. *PNAS* **107**, 5664-5668 (2010).

17 Cocker, J. *et al. Oakleaf:* an *S* locus-linked mutation of *Primula vulgaris* that affects leaf and flower development. *New Phytologist* **208**, 149-161 (2015).

18 Slater, G. & Birney, E. Automated generation of heuristics for biological sequence comparison. *BMC Bioinformatics* **6**, 31 (2005).

19 Finn, R. D. *et al.* Pfam: the protein families database. *Nucleic Acids Research* **42**, D222-D230 (2014).

20 Eddy, S. R. A new generation of homology search tools based on probabilistic inference. *Genome informatics. International Conference on Genome Informatics* **23**, 205-211 (2009).

21 Camacho, C. *et al.* BLAST+: architecture and applications. *BMC Bioinformatics* **10**, 1-9 (2009).

22 Stanke, M., Diekhans, M., Baertsch, R. & Haussler, D. Using native and syntenically mapped cDNA alignments to improve *de novo* gene finding. *Bioinformatics* **24**, 637-644 (2008).

23 Wu, T. D. & Watanabe, C. K. GMAP: a genomic mapping and alignment program for mRNA and EST sequences. *Bioinformatics* **21**, 1859-1875 (2005).

24 Altschul, S. F., Gish, W., Miller, W., Myers, E. W. & Lipman, D. J. Basic local alignment search tool. *J Mol Biol* **215**, 403-410 (1990).

25 Hallab, A. *Protein Function Prediction Using Phylogenomics, Domain Architecture Analysis, Data Integration, and Lexical Scoring*. PhD thesis, Universitäts-und Landesbibliothek Bonn (2015).

26 Conesa, A. *et al.* Blast2GO: a universal tool for annotation, visualization and analysis in functional genomics research. *Bioinformatics* **21**, 3674-3676 (2005).

27 Jones, P. *et al.* InterProScan 5: genome-scale protein function classification. *Bioinformatics* **30**, 1236-1240 (2014).

28 Trapnell, C. *et al.* Differential gene and transcript expression analysis of RNA-seq experiments with TopHat and Cufflinks. *Nature Protocols* **7**, 562-578 (2012).

29 Trapnell, C. *et al.* Differential analysis of gene regulation at transcript resolution with RNA-seq. *Nature Biotechnology* **31**, 46-53 (2013).

30 Kim, D., Langmead, B. & Salzberg, S. L. HISAT: a fast spliced aligner with low memory requirements. *Nat Meth* **12**, 357-360 (2015).

31 Pertea, M. *et al.* StringTie enables improved reconstruction of a transcriptome from RNA-seq reads. *Nat Biotech* **33**, 290-295 (2015).

32 Love, M. I., Huber, W. & Anders, S. Moderated estimation of fold change and dispersion for RNA-seq data with DESeq2. *Genome biology* **15**, 550 (2014).

33 Li, H. Aligning sequence reads, clone sequences and assembly contigs with BWA-MEM. *arXiv* **1303.3997** (2013).

34 Li, H. *et al.* The Sequence Alignment/Map format and SAMtools. *Bioinformatics* **25**, 2078-2079 (2009).
